# Kinetically Assisted Chemical Removal of Organic Contaminants by Reactive Oxygen Species: Insights from ReaxFF Molecular Dynamics Simulations

**DOI:** 10.3390/molecules30194010

**Published:** 2025-10-07

**Authors:** Zixu Wang, Yuhai Li, Peng Zhang, Fei Wang, Laixi Sun, Qingshun Bai, Mingzhi Zhu, Baoxu Wang

**Affiliations:** 1 Institute of Systems Engineering, China Academy of Engineering Physics, Mianyang 621999, China; 2 Laser Fusion Research Center, China Academy of Engineering Physics, Mianyang 621900, China; 3 School of Mechatronics Engineering, Harbin Institute of Technology, Harbin 150001, China; 4 Chongqing Research Institute, Harbin Institute of Technology, Chongqing 401135, China

**Keywords:** plasma cleaning, physical bombardment, reaction mechanism, reactive molecular dynamics simulation

## Abstract

Organic contaminants on optical components critically impair intense laser systems. Oxygen plasma cleaning is a promising non-contact method, yet the mechanism by which the initial kinetic energy of reactive oxygen species assists chemically driven removal remains unclear. This study employs ReaxFF molecular dynamics to elucidate how reactive oxygen species chemically decompose dibutyl phthalate and how kinetic energy assists chemical reactions by enhancing transport, penetration, and energy transfer. While the core removal mechanism is chemical, kinetic energy promotes plasma-contaminant encounters and facilitates access to otherwise sluggish pathways. The results show that kinetic energy is a key promoter that enhances chemical decomposition, with the contaminant decomposition rate enhanced by up to 1310% and residues reduced by 81.13% compared to pure chemical reactions. This study identifies and quantifies two dominant reaction pathways (butyl chain cleavage & benzene ring cleavage). The analysis of diffusion and energy transfer reveals that higher kinetic energy improves reactive oxygen species transport, enables deeper penetration, and selectively activates specific reaction pathways by overcoming energy barriers. Synergy with flux, dose, and temperature is also demonstrated. This work provides atomic-level insights into kinetic promotion mechanisms, supporting optimized plasma cleaning processes and contributing to the performance stability and operational longevity of intense laser systems.

## 1. Introduction

As a significant direction in advanced optoelectronic technology, the intense laser facility is a vital research tool in developing scientific fields such as inertial confinement fusion and industrial manufacturing [1,2,3]. It is characterized by high energy density, high precision, and controllability, which, in turn, places stringent demands on the surface quality and cleanliness of its optical components [4,5]. After prolonged operation within an intense laser facility, the surfaces of optical components are often contaminated by organic contaminants from manufacturing and assembly processes and material outgassing, even when in a vacuum environment [6,7]. These organic contaminants absorb a substantial amount of laser energy at specific wavelengths, inducing localized thermal explosions and damage to the optical surface, thus significantly reducing the laser-induced damage threshold (LIDT) of the optical components and limiting the system’s performance [8,9]. Plasma cleaning has become a highly efficient technical solution with considerable potential for resolving this critical issue that limits the system’s output and stable operation [10,11,12]. Compared to other common cleaning methods (such as wet and dry cleaning), plasma cleaning provides a non-contact, in situ implementable method to remove organic contaminants from optical surfaces [13,14,15]. The technology possesses advantages such as high removal efficiency and controllable damage, allowing for the periodic restoration of component surface cleanliness without the need for the complex, time-consuming disassembly of the laser system, thereby ensuring the long-term, high-performance operation of the intense laser facility [16,17,18,19].

Traditional experimental characterization techniques such as X-ray Photoelectron Spectroscopy (XPS), Scanning Electron Microscopy (SEM), and Atomic Force Microscopy (AFM) can perform ex situ surface analysis, effectively characterizing surface composition and morphology before and after plasma treatment [20,21]. However, due to the lack of necessary temporal resolution, the ultrafast chemical bond breaking and formation that occur during plasma bombardment, as well as the intermediate products and reaction pathways that determine cleaning efficiency, are difficult to capture through direct experimental observation [22,23]. Owing to the high reactivity of plasma, its interaction with surface organic contaminants occurs at an extremely fast reaction rate and on an extremely short timescale (femtoseconds to picoseconds), with intermediate products and reactive oxygen groups exhibiting extremely short lifetimes during the reaction process. Consequently, traditional experimental characterization methods, which can only characterize the final reaction products, are unable to monitor species transformation and reaction pathways in real-time during the cleaning process [24,25]. In situ emission spectroscopy partially addresses this issue, but the reaction information it provides is limited, and it cannot analyze the kinetic-promotion effect (due to the ROS impact on energy) on chemically driven removal [26,27]. Secondly, traditional experimental methods struggle to decouple the synergistic effects between physical bombardment (driven by kinetic energy transferring) and chemical reactions (generated by reactive oxygen groups) in plasma-surface organic contaminant interactions. This means it is impossible to conduct quantitative analysis on the respective contributions of chemical reaction and kinetic assistance in the cleaning reaction, thereby hindering the effective optimization of process parameters for cleaning protocols and leading to issues such as insufficient cleaning or substrate damage from over-cleaning. For example, AFM images can reveal the reduction in surface roughness of GaAs samples after plasma pretreatment, but this alteration in surface morphology results from a combination of effects, making it difficult to directly differentiate the specific contributions of chemical reactions and physical bombardment through AFM [28]. Furthermore, traditional characterization methods cannot precisely determine the penetration depth or collision reactions of reactive oxygen groups, nor can they elucidate the reaction process of the layer-by-layer decomposition of organic contaminants, which further impedes in-depth research into the microscopic mechanisms of plasma cleaning reactions [29]. These experimental limitations severely restrict the investigation of the fundamental mechanisms governing the reactions between reactive oxygen groups and organic contaminants during plasma cleaning, particularly concerning the relative contributions of kinetic energy transfer and chemical reactions, the analysis of energy thresholds for bond dissociation and reaction pathway selection, and the study of the influence of kinetic energy transfer on cleaning efficiency. In the absence of understanding microscopic reaction mechanisms, the optimization and development of plasma cleaning technology remain constrained by empirical experimentation rather than being guided by a first-principles theoretical understanding.

To address the gaps in mechanistic studies, molecular dynamics simulations employing the ReaxFF reactive force field have become an important tool for investigating the interactions between plasma and surface organic contaminants. ReaxFF is a simulation method that combines the advantages of quantum mechanics and traditional molecular dynamics based on bond-order, thereby enabling the description of bond breaking and formation between molecules in complex systems [30]. In recent years, the application of ReaxFF in studies of plasma-surface interactions has become increasingly widespread [31,32]. Sun et al. explained the surface polishing and subsurface defect removal mechanisms of single-crystal silicon etched by fluorine atom plasma through ReaxFF molecular dynamics simulations [33]. Yin et al. utilized ReaxFF molecular simulation techniques to study the interaction mechanisms between O atoms, OH radicals, and H_2_O_2_ molecules and cellulose, providing fundamental insights into the atomistic-level interaction mechanisms and guidance for practical experiments [34]. Concurrently, ReaxFF molecular dynamics simulations can also be used to study the physical effects of atomic collisions, enabling a comprehensive investigation of reaction mechanisms under coupled physical and chemical effects, as well as decoupling the physical and chemical effects by adjusting system parameters. Zhou et al. utilized reactive molecular dynamics to simulate the collision and erosion processes of atomic oxygen on composite materials, investigating the product characteristics and reaction pathways of the material’s surface layer, as well as the microscopic mechanisms of layer cracking [35]. Kim et al. performed etching molecular dynamics simulations using ReaxFF parameters optimized for Si/O/H/F systems, investigating the removal mechanisms and products of clusters, and observed changes in reaction initiation speed and dissociation fraction at different incident energies [36]. Zhang et al. accurately described the molecular structure of 3,4-Dinitrofurazanfuroxan (DNTF) after re-optimizing ReaxFF parameters and simulated the reaction mechanism of DNTF under shock loading, clarifying the main decomposition pathways of the material under impact [37]. However, in the study of the reaction mechanisms of plasma cleaning of organic contaminants on optical component surfaces, gaps remain regarding how initial kinetic energy promotes chemistry to influence removal rates and pathway selection. The energy threshold dependence of different reaction pathway selections and different chemical bond breaking has not yet been elucidated. Due to the lack of decoupling between the physical and chemical effects of oxygen plasma on organic contaminants, the proportional contribution of kinetic energy bombardment in the cleaning reaction remains unclear, and there is a lack of quantitative analysis on how changes in incident kinetic energy affect the progression of the cleaning reaction.

Therefore, this work aims to systematically elucidate the fundamental mechanism by which the kinetic energy of reactive oxygen species (ROS) governs the reaction pathway selection and cleaning efficiency in plasma-based removal of organic contaminants, utilizing ReaxFF-based molecular dynamics simulations with dibutyl phthalate (DBP) as a representative model pollutant. Through quantitatively controlling parameters including initial kinetic energy, flux, irradiation dose, and ambient temperature, we disentangle kinetic assistance from chemical reactions (chemistry-only control vs. kinetically assisted case) and trace the dynamic evolution of reaction intermediates and pathways at the atomic level. This dramatic enhancement is achieved through multiple mechanisms: enabling deep penetration into the contaminant layer, selectively activating specific bond cleavage sequences (C-O and C-C fission), efficiently transferring energy to elevate system potential above critical reaction barriers, and exhibiting strong synergistic effects with other plasma parameters. These insights not only deepen the fundamental understanding of energy-field-assisted surface reaction dynamics but also provide atomistic-level guidance for optimizing plasma cleaning protocols, ultimately contributing to the achievement of high-efficiency, damage-free surface restoration for precision optical components operating in intense laser systems. In this work, we treat ROS–DBP chemistry as primary. The assigned translational energies of incident neutral O serve as a proxy for sheath-accelerated impacts that promote removal by increasing encounter frequency and transiently assisting activation. This framing enables us to decouple kinetic assistance from chemical reactivity and to assess how kinetic parameters modulate pathway access and desorption.

## 2. Results

### 2.1. Analysis of Typical Reaction Pathways and Products

Multiple reaction pathways exist in the chemical processes during plasma cleaning, with the primary reaction process involving the stepwise decomposition of DBP into small molecular groups by ROS. Therefore, different reaction pathways during the decomposition can be characterized by their corresponding signature products. First, a comparative analysis of the reaction products between the zero-kinetic energy group (initial kinetic energy of 0.0083 eV) and the standard kinetic energy experimental group (initial kinetic energy of 75 eV) was conducted to illustrate the typical reaction pathways and their characteristic products, thereby laying the groundwork for subsequent analysis of the influence of kinetic energy on reaction pathways and rates.

**Figure 1:**
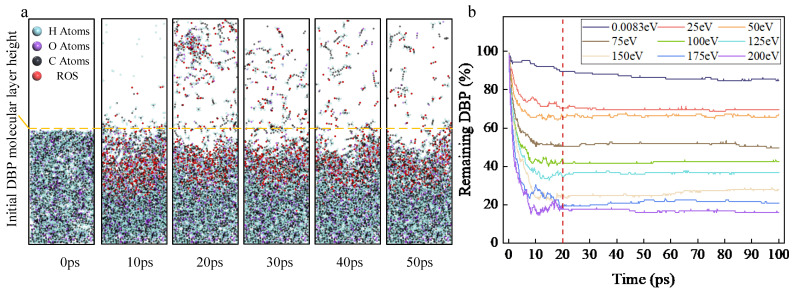
(**a**) Schematic diagram of the removal process of dibutyl phthalate (DBP) via its reaction with reactive oxygen species (ROS); (**b**) the variation in DBP residue ratio with kinetic energy and time. The red line in the figure indicates that all ROS were inserted into the simulation box at 20 ps, and no new ROS entered the simulation.

Figure 1a shows the variation in the simulation process of the reaction between ROS and DBP over time at 75 eV. It can be seen that in the early stage of the reaction (10 ps), some DBP molecules are decomposed, and the height of the DBP molecular layer decreases significantly. In the later stage of the reaction, a large number of DBP molecules decomposed and desorbed, and the height of the molecular layer of organic pollutants decreased significantly. Figure 1b shows the variation in the DBP residue ratio over time under different initial kinetic energies. The DBP residue ratio is defined as the ratio of the number of DBP molecules remaining in the simulation system at the current time to the initial number of DBP molecules in the system. According to the standard experimental parameters (ROS insertion interval of 40 fs, insertion number of 500), all reactive oxygen species are completely inserted 20 ps after the reaction starts. Between 0–10 ps, the DBP residue ratio decreases significantly, while from 10–20 ps, the decrease slows down. This indicates that intense decomposition occurs within 0–10 ps, the decomposition rate gradually decreases from 10–20 ps, and after 20 ps, the DBP decomposition reaction progressively stops, approaching a steady state.

Since the initial stage of the reaction involves the first contact between ROS and DBP, characteristic products of the reaction pathways are abundant, making the pathway identification more observable. In the later stages, intermediate products are further decomposed, interfering with the observation of reaction pathways. Therefore, 5 ps, 10 ps, 15 ps and 20 ps were selected as observation windows for analyzing reaction pathways and main products.

The species generation in the kinetic energy experiments was analyzed. The proportion of a specific product molecule relative to the total number of molecules in the system was calculated, and the product with the highest proportion was selected as the main observed product. By comparing with the visualized reaction process shown in Figure 1a, C_6_-based products (chemical formula C_6_H_x_O_y_), C_8_-based products (C_8_H_x_O_y_), and C_12_-based products (C_12_H_x_O_y_) were identified as characteristic products of the main reaction pathways. Additionally, since actual cleaning devices operate in vacuum systems where small molecules generated from the complete decomposition of DBP are pumped away, the count of all small molecules (those with three or fewer carbon atoms per molecule, excluding ROS) was used as an indicator of the complete decomposition of the reactants.

**Figure 2:**
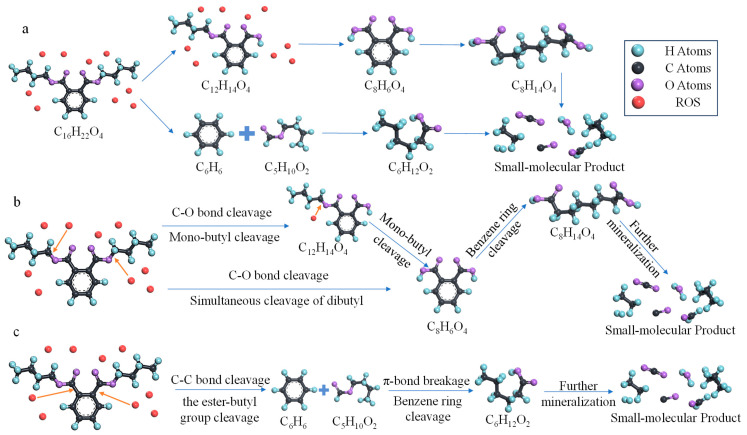
Roadmap of the DBP removal reaction: (**a**) Schematic illustration of the two main reaction pathways; (**b**) step-by-step reaction pathway of mono-butyl cleavage and simultaneous dual-butyl cleavage; and (**c**) detailed reaction pathway of ester group cleavage.

Based on the analysis of the main products and reaction processes, it can be concluded that the decomposition of DBP by reactive oxygen species (ROS) in the plasma primarily follows two types of reaction pathways, as illustrated in Figure 2a. The first type of reaction pathway, shown in Figure 2b, involves the initial cleavage of a C-O single bond in DBP, leading to the dissociation of a butyl side chain. This is followed by π-bond breakage, resulting in benzene ring cleavage, and finally deep degradation into small molecular species. Within this pathway, two distinct stepwise processes exist: firstly, DBP undergoes mono-butyl cleavage, generating characteristic C_12_-based products, followed by cleavage of the other butyl side chain; secondly, simultaneous cleavage of both butyl side chains on the DBP molecule occurs, producing characteristic C_8_-based products. The second type of reaction pathway, depicted in Figure 2c, involves the breakage of a C-C single bond, leading to cleavage of the ester-butyl group, which generates characteristic C_6_-based aromatic products. This is followed by π-bond breakage and benzene ring cleavage, ultimately resulting in deep degradation into small molecules. Therefore, by analyzing the yields of different characteristic products, the proportional contribution of each reaction pathway can be correspondingly determined, thereby facilitating the analysis of the influence of initial kinetic energy on the selection of reaction pathways.

### 2.2. The Core Influence of the Initial Kinetic Energy and Other Parameters of Reactive Oxygen Species on the Cleaning Reaction

Having understood the specific pathways and characterization methods for the decomposition reaction by which ROS in the plasma remove DBP, we can proceed to quantitatively analyze the extent of the influence of the initial kinetic energy of the plasma on the selection of reaction pathways and the cleaning efficiency.

As shown in Figure 1a, when the initial kinetic energy is zero, the DBP removal rate is relatively uniform between 0–100 ps, with decomposition reactions continuously occurring as the ROS diffuse and come into contact with DBP; the average reaction rate is 0.48 s^−1^ (number of DBP decompositions per unit time). After imparting initial kinetic energy to the ROS, the ROS are uniformly deposited on the surface of the organic contaminant layer within 0–20 ps. Compared to the zero-kinetic-energy group, it is evident that the DBP decomposition reaction predominantly occurs within 0–20 ps, and as the initial kinetic energy increases, the DBP decomposition rate (2.43, 2.62, 4.05, 4.61, 5.06, 5.76, 6.34, and 6.70 s^−1^, respectively) rises rapidly. Even with the minimum initial kinetic energy (25 eV), the DBP decomposition rate increased by 406.3% compared to the zero-kinetic-energy group. At the maximum DBP decomposition rate (maximum initial kinetic energy of 200 eV), it increased by 1310% relative to the zero-kinetic-energy group.

In the zero-kinetic-energy group, upon completion of the cleaning reaction, the residue ratio of DBP was 84.8%. After imparting initial kinetic energy to the ROS, the DBP residue ratio decreased rapidly with increasing initial kinetic energy (69.6%, 67.2%, 49.6%, 42.4%, 36.8%, 28%, 20.8%, and 16%, respectively). Compared to the zero-kinetic-energy group, even with the minimum initial kinetic energy, the DBP residue ratio decreased by 17.92%, while at the lowest DBP residue ratio (maximum initial kinetic energy of 200 eV), it decreased by 81.13%. This comparative data indicates that ROS with initial kinetic energy contacts DBP more rapidly, inducing its decomposition reaction, and can substantially enhance the DBP decomposition rate and removal efficiency.

Knowing the significant promoting effect of the initial kinetic energy of ROS on the DBP decomposition reaction, the next step is to define the mechanism by which initial kinetic energy influences the cleaning reaction. To more accurately quantify the factors affected by initial kinetic energy, the instantaneous abundance is first defined: for any target species in the reaction system, the instantaneous abundance is recorded as the ratio of the number of molecules of the target species at time *t* with incident kinetic energy *E* to the initial number of DBP molecules in the system (the normalization reference), used to represent the relative conversion rate. This data can quantify the dominance of a reaction route and, through normalization, eliminates statistical drift caused by system expansion, enabling comparison of pathway weights under different initial kinetic energies.

Furthermore, it is noted that during the reaction, ROS may combine with H_2_ or hydrogen atoms dissociated in the system or undergo further oxidation, generating various hydrogen-oxygen compounds. Therefore, the oxygen-containing fraction *ϕ*_HO_ is defined: *ϕ*_HO_ is defined as the instantaneous fraction of oxygen-bearing species among molecules containing only H and O elements. A high *ϕ*_HO_ represents the degree of secondary oxidation of hydrogen atoms or H_2_ in the system. It can measure the re-oxidation capability of oxygen radicals towards hydrogen radicals and serve as a discriminator of oxygen atom activity.

Subsequently, to assess the selective proportion among different reaction pathways, the reaction pathway weight is defined: the proportion of the number of molecules of a characteristic product of a specific reaction pathway to the total number of molecules of all characteristic products at a given time. The higher this weight coefficient, the greater the contribution of the corresponding reaction pathway in the DBP decomposition reaction. The calculation formulas for the above parameters are as follows:

(1) $$n\left(C_{12},t,E\right)=\frac{n\left(C_{12}H_{x}O_{y}\right)}{N\left(C_{16}H_{22}O_{4}\right)}$$

(2) $$n\left(C_{8},t,E\right)=\frac{n\left(C_{8}H_{x}O_{y}\right)}{N\left(C_{16}H_{22}O_{4}\right)}$$

(3) $$n\left(C_{6},t,E\right)=\frac{n\left(C_{6}H_{x}O_{y}\right)}{N\left(C_{16}H_{22}O_{4}\right)}$$

(4) $$n\left(C_{j},t,E\right)=\frac{n\left(C_{j}H_{x}O_{y}\right)}{N\left(C_{16}H_{22}O_{4}\right)}(j\leq 3)$$

(5) $$\phi_{HO}(t,E)=\frac{\sum n(H_{a}O_{b};b\geq 1)}{\sum n(H_{a}O_{b})}$$

(6) $$R_{SB}=\frac{n(C_{12})}{n(C_{12}+C_{8}+C_{6})}$$

(7) $$R_{DB}=\frac{n(C_{8})}{n(C_{12}+C_{8}+C_{6})}$$

(8) $$R_{Ph}=\frac{n(C_{6})}{n(C_{12}+C_{8}+C_{6})}$$

In the equation, *n*(C_12_, *t*, *E*) denotes the instantaneous abundance of C_12_-based products at time *t* and initial kinetic energy *E*; *n*(C_12_H*_x_*O*_y_*) represents the number of C_12_-based product molecules in the simulation system at time *t* and initial kinetic energy *E*; and *N*(C_16_H_22_O_4_) indicates the initial number of DBP molecules in the simulation system. *ϕ*_HO_(*t*, *E*) represents the oxygen-containing fraction of the system at time *t* and initial kinetic energy *E*. *R*_SB_, *R*_DB_, and *R*_Ph_ denote the weight coefficients of the reaction pathway for mono-butyl side chain cleavage, dual-butyl side chain cleavage, and benzene ring cleavage, respectively.

**Figure 3:**
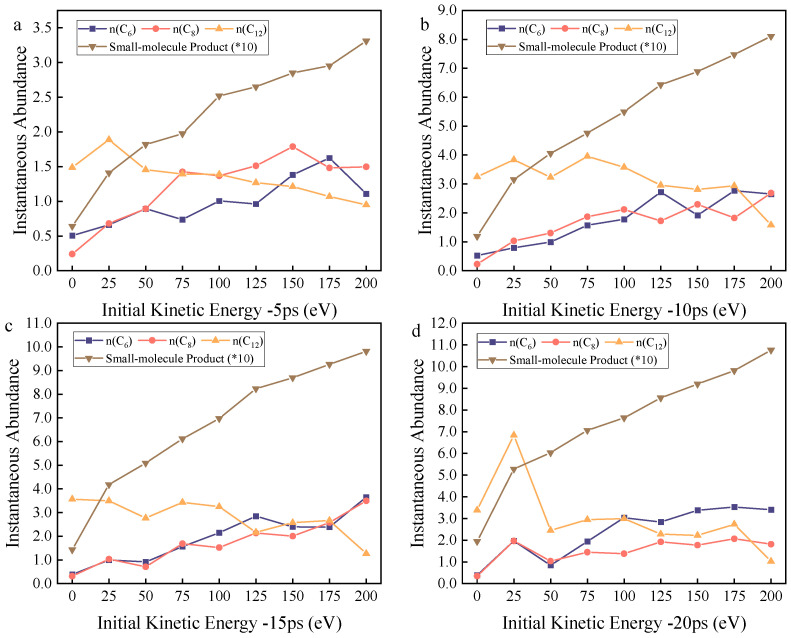
The figure illustrates the instantaneous abundance of characteristic products under varying levels of kinetic energy across different time points; (**a**) the variation in instantaneous abundance of each species with kinetic energy when the reaction time is 5 ps; (**b**) the variation in instantaneous abundance of each species with kinetic energy when the re-action time is 10 ps; (**c**) the variation in instantaneous abundance of each species with kinetic energy when the re-action time is 15 ps; (**d**) the variation in instantaneous abundance of each species with kinetic energy when the re-action time is 20 ps.

**Figure 4:**
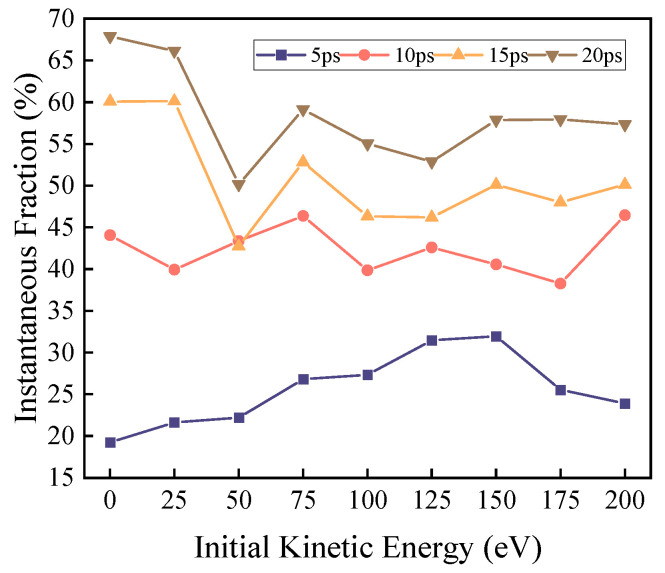
The figure illustrates the oxygen-containing fraction of the system under varying levels of kinetic energy across different time points.

**Figure 5:**
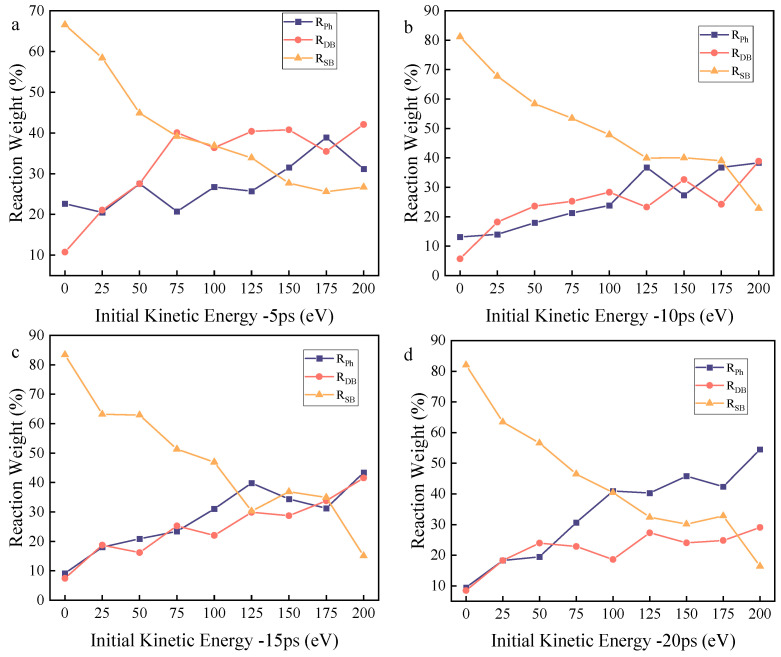
The figure illustrates the weight coefficients of the reaction pathway for mono-butyl side chain cleavage, dual-butyl side chain cleavage, and benzene ring cleavage under varying levels of kinetic energy across different time points. (**a**) The variation in the reaction weights of each path with kinetic energy when the reaction time is 5 ps; (**b**) the variation in the reaction weights of each path with kinetic energy when the reaction time is 10 ps; (**c**) the variation in the reaction weights of each path with kinetic energy when the reaction time is 15 ps; and (**d**) the variation in the reaction weights of each path with kinetic energy when the reaction time is 20 ps.

Thus, the variations in the instantaneous abundance of each characteristic product, the oxygen-containing fraction of the system, and the weight coefficients of each reaction pathway with respect to time and kinetic energy are shown in Figure 3, Figure 4 and Figure 5, respectively.

According to the experimental setup, reactive oxygen atoms are uniformly deposited within the 0–20 ps timeframe, and the DBP decomposition reaction primarily occurs within 0–20 ps. Therefore, the analysis of products and reaction pathway weights focuses mainly on the 0–20 ps period. The characteristic products corresponding to different reaction pathways are observed separately.

The zero-kinetic-energy group, which excludes the physical effects of kinetic energy, reflects the pure chemical effect of the reaction between reactive oxygen atoms and DBP and can serve as a control group. Hence, the reaction behavior of the zero-kinetic-energy group is analyzed first.

Under zero kinetic energy conditions, the weight of the mono-side chain cleavage reaction remains consistently high. Considering that the reactive oxygen atoms in the zero-kinetic-energy group require time to diffuse within the system and make extensive contact with DBP after 5 ps, the decomposition reaction occurs comprehensively. At this point, the weight of the mono-side chain cleavage reaction remains above 80%, while the weights of the dual-side chain cleavage reaction and benzene ring cleavage reaction continue to decrease. This indicates that for the zero-kinetic-energy group, the reactive oxygen atoms have low activation energy and weak oxidation capacity, resulting in incomplete DBP decomposition.

It is noted that *ϕ*_HO_ increased by 47% at 20 ps compared to 5 ps and continues to rise over time, indicating that a large number of reactive oxygen atoms are captured by free hydrogen atoms, forming stable small molecules such as H_2_O, and are unable to oxidize and decompose DBP. This indirectly demonstrates the weak oxidation capacity of reactive oxygen atoms in the absence of kinetic energy.

The yield of C_12_ exhibits a single peak at t = 5 ps, with the peak located around 25 eV. Meanwhile, its yield decreases with increasing kinetic energy, and the weight of the mono-side chain cleavage reaction pathway continuously decreases as kinetic energy rises. The peak yield is primarily due to the fact that even with a very low initial kinetic energy (25 eV) imparted to the reactive oxygen atoms, the reaction rate is significantly increased compared to the zero-kinetic-energy group. Thus, the C_12_ generation rate of the 25 eV group increased by 26.8% compared to the zero-kinetic-energy group within the same timeframe. As kinetic energy further increases, the weight of the mono-side chain cleavage reaction decreases, leading to a decline in C_12_ yield.

The yield of C_8_ shows a single peak at t = 5 ps, with the peak located around 150 eV. Meanwhile, its yield initially increases and then decreases with rising kinetic energy. The weight of the dual-side chain cleavage reaction pathway generally exhibits an increasing trend with increasing kinetic energy, reaching a plateau at 75 eV and subsequently maintaining fluctuations. At this point, this reaction pathway approaches saturation.

The yield of C_6_ displays a single peak at t = 5 ps, with the peak located around 175 eV. Meanwhile, its yield initially increases and then decreases with rising kinetic energy. The weight of the benzene ring cleavage reaction pathway generally shows an increasing trend with increasing kinetic energy, fluctuating within the 125–200 eV range, while increasing overall.

The yield of small molecules continuously increases over time at the same time point. The oxygen-containing fraction *ϕ*_HO_ gradually increases within the 0–150 eV range at the same time point, peaks at 150 eV, and then gradually decreases. Under the same kinetic energy (any kinetic energy), *ϕ*_HO_ continuously increases over time.

Analysis of the simulation results indicates that the initial kinetic energy of the ROS does not linearly enhance all decomposition pathways. Instead, it selectively activates different reaction channels by overcoming the energy barriers of various reaction pathways, governing the entire process from initial cleavage to deep decomposition of DBP molecules. Overall, an activation sequence of mono-butyl side chain cleavage → dual-butyl side chain cleavage → direct aromatic ring cleavage is observed. This suggests that as the initial kinetic energy of ROS increases from 0.0083 eV to 200 eV, the reaction system sequentially surmounts three increasing reaction energy barriers. The specific reaction pathway analysis is as follows.

First, the variation in the instantaneous abundance of C_12_ (characteristic product of mono-butyl side chain cleavage) with time and initial kinetic energy indicates that initial kinetic energy within a relatively low range (25 eV) can significantly promote mono-butyl side chain cleavage (compared to the zero-kinetic-energy group). At this stage, this reaction pathway is the most sensitive to kinetic energy and is activated first. This may originate from the spatial exposure of the butyl side chain, which has minimal shielding and undergoes strong collisions first. Moreover, the C-O single bond connecting the ester group and the butyl group is relatively weak, making it the most vulnerable point for cleavage under low-energy perturbations. The aromatic ring skeleton, located inward within the molecule and surrounded by a strong electron cloud, requires higher kinetic energy to provide sufficient energy transfer for the reaction to occur. Thus, kinetic energy significantly increases the collision efficiency between ROS and the C-O single bond near the ester group, overcoming the reaction energy barrier and more readily initiating oxidative cleavage. However, as kinetic energy further increases (25–200 eV), the C_12_ yield continuously decreases, and its reaction weight declines within the 0–200 eV range. This phenomenon indicates that at higher initial kinetic energies, other reaction pathways are activated, leading to a shift in reaction pathways: mono-side chain cleavage is replaced by other pathways, such as simultaneous dual-side chain cleavage or direct benzene ring cleavage. Furthermore, as initial kinetic energy increases, the activity of ROS further enhances, and C_1_2 products are further decomposed, unable to exist stably as reaction intermediates. Consequently, their weight and current yield decrease. The changes in the instantaneous abundance and reaction weight of C_12_ products vividly demonstrate the catalytic effect of physical action on specific chemical reactions.

Second, the instantaneous abundance of C_8_ (characteristic product of dual-butyl side chain cleavage) generally shows an increasing trend within the 100–150 eV kinetic energy range, indicating that increased kinetic energy effectively promotes dual-side chain cleavage. The corresponding trend in reaction weight further demonstrates that within the 0–75 eV range, the dual-side chain cleavage pathway is effectively activated in this kinetic energy interval and gradually becomes the dominant process as initial kinetic energy increases. Compared to the mono-side chain cleavage pathway, the dual-side chain cleavage pathway has a higher energy barrier, thus requiring higher initial kinetic energy for activation. Moreover, compared to low-kinetic-energy conditions, higher kinetic energy results in stronger ROS activity, which does not diminish after reacting with a single side chain. After the initial kinetic energy exceeds 75 eV, the reaction weight reaches a plateau, indicating that the dual-side chain cleavage pathway saturates beyond 75 eV. This may be because increased kinetic energy only promotes a higher overall decomposition reaction rate without further enhancing the selectivity of this pathway, while the benzene ring cleavage reaction is activated, leading to a balanced reaction weight for dual-side chain cleavage. The changes in the instantaneous abundance and reaction weight of C_8_ products illustrate that as kinetic energy increases, higher energy input drives the reaction pathway toward more intense and thorough decomposition. Simultaneously, the physical impact of initial kinetic energy makes the molecular structure of DBP more susceptible to destruction, which is difficult to achieve with pure chemical etching in the zero-kinetic-energy group.

Third, the instantaneous abundance of C_6_ (characteristic product of direct benzene ring cleavage) generally increases and exhibits fluctuations within the 0–175 eV kinetic energy range. This reflects the dynamic competition among reaction pathways. The slight decreases in reaction weight at 75 eV and 125 eV may correspond to the peak of dual-butyl side chain cleavage, where dual-side chain cleavage consumes some ROS, reducing the probability of direct reaction with the benzene ring. After 125 eV, the weight of the benzene ring cleavage pathway increases and reaches its peak. This indicates that higher initial kinetic energy significantly promotes direct benzene ring cleavage, making it an important deep decomposition pathway. This is because the benzene ring, due to its conjugated system, is highly stable and difficult to directly cleave at low and medium kinetic energies. Thus, higher energy is required to break the aromatic ring. Increased kinetic energy enables ROS to effectively insert into π-bonds, causing ring opening and generating ring-opening products. This trend is consistent with the mechanism of high-energy particle-induced aromatic ring cleavage, where the kinetic energy of ROS helps overcome steric hindrance and electron cloud repulsion. This clearly demonstrates that high-kinetic-energy physical bombardment is an effective means to overcome high reaction energy barriers and achieve complete decomposition of contaminants.

Small molecule products, as characteristic indicators of the complete decomposition of organic contaminants, show a continuous increase in instantaneous abundance over time, consistent with the gradual nature of oxidative decomposition. Regardless of the initial kinetic energy, the continuous generation of small molecules indicates that DBP decomposition ultimately tends toward complete decomposition. Once larger product fragments are generated, they continue to break down into subsequent chain oxidation processes, producing final products such as CO_2_ and H_2_O. Increased initial kinetic energy only enhances the generation rate and quantity of small molecules without involving the selection of reaction pathways. This indicates that regardless of the intermediate pathway chosen, higher energy input will eventually lead to decomposition into small molecules, and increasing the initial kinetic energy of ROS remains an effective way to achieve complete decomposition of organic contaminants.

The variation of *ϕ*_HO_ provides a measure of the system’s oxidation degree. At the same time point, *ϕ*_HO_ gradually increases within the 0–150 eV kinetic energy range, peaks at 150 eV, and then gradually decreases. This indicates that increased initial kinetic energy facilitates oxidation reactions. The oxidative decomposition of DBP is essentially a dehydrogenation and oxidation process. High-kinetic-energy ROS possesses higher collision energy, more effectively breaking C-H bonds in molecules for dehydrogenation, and the detached hydrogen atoms rapidly combine with reactive oxygen. Moreover, H_2_O and H_2_O_2_ are typically final products of deep oxidation, and their increased proportion indicates that the reaction is progressing toward complete decomposition. The decrease in ϕ_HO_ after further increases in kinetic energy may be due to overly intense decomposition reactions caused by high-energy collisions, generating a large number of hydrogen-rich free radicals and producing H_2_. Meanwhile, ROS are further captured by DBP molecules and react, leading to a decrease in the oxygen-containing proportion. The continuous increase in ϕ_HO_ over time at the same kinetic energy also illustrates that DBP decomposition ultimately tends toward complete decomposition, with released H atoms continuously reacting with ROS to form H_2_O and H_2_O_2_.

The aforementioned research has confirmed that the initial kinetic energy of ROS serves as a key physical parameter playing a decisive role in the degradation process of DBP. However, in actual plasma cleaning processes, the reaction is coordinately regulated by plasma parameters (flux, irradiation dose N_ROS_) and environmental parameters (ambient temperature). To systematically reveal the multi-parameter synergistic mechanism, this study investigates the quantitative relationship between irradiation flux, irradiation dose, ambient temperature, and the efficiency and extent of DBP removal, exploring how these parameters synergize with the initial kinetic energy of ROS to influence the decomposition reaction efficiency.

**Figure 6:**
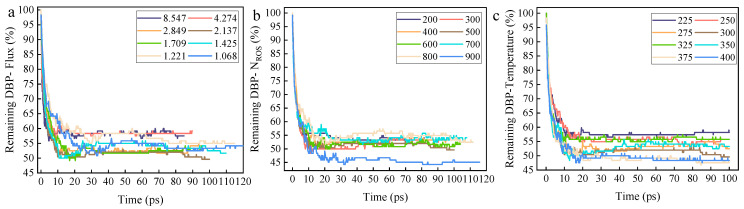
The figure illustrates the variation in the DBP Remaining ratio over time under different plasma parameters (plasma irradiation flux, plasma irradiation dose (N_ROS_), and ambient temperature). (**a**) The different curves correspond to eight numerical gradients for irradiation flux of 8.547 × 10^26^ cm^−2^s^−1^, 4.274 × 10^26^ cm^−2^s^−1^, 2.849 × 10^26^ cm^−2^s^−1^, 2.137 × 10^26^ cm^−2^s^−1^, 1.709 × 10^26^ cm^−2^s^−1^, 1.425 × 10^26^ cm^−2^s^−1^, 1.221 × 10^26^ cm^−2^s^−1^, and 1.068 × 10^26^ cm^−2^s^−1^. (**b**) The different curves correspond to eight numerical gradients for irradiation doses of 200, 300, 400, 500, 600, 700, 800, and 900. (**c**) The different curves correspond to eight numerical gradients for ambient temperatures of 225 K, 250 K, 275 K, 300 K, 325 K, 350 K, 375 K, and 400 K.

First, according to the selected experimental design, a stepped experiment with eight gradients was conducted for each characteristic parameter. The DBP residue ratio for each group was calculated, and the data are plotted in Figure 6a–c. Data analysis reveals that the final residue ratio of DBP initially decreases and then increases with rising irradiation flux, while it decreases approximately monotonically with increasing irradiation dose and ambient temperature.

The non-monotonic change in DBP residue with irradiation flux—first decreasing and then increasing—indicates that there exists an optimal window of irradiation flux for enhancing DBP removal rate. In the lower flux range, increasing the flux significantly improves the coverage of active sites per unit time and the advancement rate of the reaction front. Combined with the deep penetration capability of high-kinetic-energy ROS, this accelerates the reaction through a spatiotemporal synergistic effect: high-kinetic-energy ROS are responsible for in-depth breakthrough, while high flux ensures sufficient active particles to fill and clean the opened reaction space, thereby significantly reducing the residue. However, when the flux exceeds the optimal value, the residue increases instead of decreasing. This may stem from surface passivation or competitive consumption induced by over-irradiation. An excessively high flux may cause a sharp increase in the concentration of ROS in the surface region, potentially leading to self-recombination reactions between ROS, resulting in the ineffectual consumption of active species; high-frequency particle bombardment may cause excessive cross-linking or carbonization of surface organic matter, forming a dense inert carbon layer that blocks contact between internal DBP and ROS.

Secondly, irradiation dose exhibits a monotonic negative correlation with DBP residue, reflecting the cumulative effect of reaction energy input. Regardless of the kinetic energy of individual ROS, an increase in the total dose directly implies an increase in total energy input and the total number of active particles. In the initial stage of the reaction, ROS first react with molecules on the surface of the organic contaminant layer, while the high initial kinetic energy of ROS allows them to penetrate the DBP molecular layer, forming reaction channels. Subsequently, sustained irradiation with a sufficient number of ROS utilizes these reaction channels to continue advancing into the interior of the DBP molecular layer, reacting with a larger area of DBP molecules to complete the cleaning reaction and significantly reduce the DBP residue ratio. The irradiation dose ensures the thoroughness of the reaction, effectively complementing the reaction rate promoted by kinetic energy.

Finally, an increase in ambient temperature monotonically promotes DBP removal, indicating a positive synergistic effect between thermal effects and particle kinetic energy effects. Firstly, the temperature rise provides an additional thermodynamic driving force for all reaction pathways (especially the high-energy-barrier benzene ring opening reaction), reducing the effective energy barrier that ROS need to overcome, enabling even ROS with relatively low kinetic energy to initiate more profound decomposition reactions. Secondly, the temperature increase significantly amplifies the thermal motion amplitude of DBP molecular chains, promoting the desorption and diffusion of reaction products and preventing them from blocking the reaction interface. Therefore, the coupling of the thermal kinetic energy provided by the ambient temperature and the initial kinetic energy of ROS collectively determines the effective energy per collision, more effectively exciting the reaction pathways.

The aforementioned simulation results can also be compared and validated against our team’s previous experimental findings [10,17,22]. Experimentally, it was observed that the decomposition rate and efficiency of DBP significantly improved with the optimization of plasma power, yet the underlying mechanism behind how power enhancement influences the decomposition process could not be elucidated. Molecular dynamics simulations similarly reflect this trend and, moreover, provide a rational analysis demonstrating that the kinetic energy of plasma is a critical factor affecting the decomposition reaction. Experimentally, even after cleaning, residual carbon signals were detected on the sample surface by XPS, which can be attributed to stable aromatic intermediates. The simulations reveal that cleavage of the benzene ring requires a higher energy barrier compared to side-chain cleavage. The simulation results thereby explain why complete carbon removal is so challenging in experiments. Similarly, the variation in spectral excitation intensity across different measurement regions and differing irradiation times in experiments corresponds, respectively, to variations in plasma flux and irradiation dose in the simulations, and their influence on the DBP removal rate and efficiency follows identical trends. Thus, although certain parameters in the molecular dynamics simulations were scaled to ensure computational feasibility, the consistency in trends between the simulation and experimental outcomes supports the reliability of the model.

### 2.3. Analysis of the Reaction Mechanism of ROS Based on Diffusion and Energy Characteristics

The aforementioned research results indicate that the initial kinetic energy of ROS is a key physical parameter governing the selectivity of their reaction pathways with organic contaminants. However, the influence of kinetic energy does not exist in isolation; the final macroscopic reaction efficiency and selectivity are the microscopic outcome of the combined effects of the transport and diffusion behavior of ROS within the contaminant matrix and the local reaction energy. To deeply reveal the essence of how initial kinetic energy influences the reaction mechanism, this section will further integrate the mean square displacement (MSD) of oxygen plasma components, the penetration depth, and the evolution of the system’s kinetic and potential energy. From the perspectives of diffusion dynamics and system energy distribution, a comprehensive analysis of the intrinsic relationship between the transport capacity of ROS, their energy state, and the ultimate reaction efficiency and pathway selection will be conducted.

**Figure 7:**
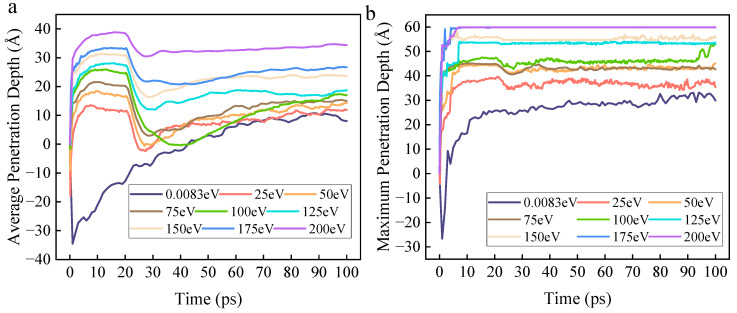
The figure shows the relationship between the average penetration depth and the maximum penetration depth with time and initial kinetic energy, and both subfigures share the same legend. (**a**) The average penetration depth is the average depth that all existing ROS particles penetrate into the DBP molecular layer. (**b**) The maximum penetration depth is the maximum depth that all existing ROS particles can penetrate into the DBP molecular layer.

Firstly, based on atomic coordinates, the penetration depth of ROS into the organic contaminant molecular layer can be calculated. The relationships of the average penetration depth and the maximum penetration depth with time and initial kinetic energy are shown in Figure 7a,b, respectively. Since ROS are incident from a fixed height above the DBP molecular layer, the penetration depth is negative at the beginning of the reaction. The data results show that the average penetration depth of the zero-kinetic-energy group increases from 0 to 100 ps and eventually stabilizes. For the groups with kinetic energy, the average penetration depth increases rapidly within 0–10 ps, enters a plateau phase between 10–20 ps, then decreases rapidly, and finally increases slowly toward a steady state. Moreover, as the kinetic energy increases, the average penetration depth gradually increases. The maximum penetration depth increases progressively over time and with increasing kinetic energy.

Specifically, the average penetration depth of the zero-kinetic-energy group increases monotonically with time and eventually stabilizes. This is because the initial kinetic energy of zero causes ROS to rely primarily on concentration gradient-driven, relatively slow random diffusion until they achieve a uniform distribution in the simulation system, resulting in limited penetration capability into the organic contaminant molecular layer. In stark contrast, the average penetration depth of the groups with initial kinetic energy exhibits a complex pattern characterized by “rapid rise-plateau-decline-slow increase.” Within the 0–10 ps range, high-kinetic-energy ROS utilize their initial kinetic energy to overcome intermolecular forces and directionally penetrate the DBP molecular layer, with higher kinetic energy leading to greater penetration depth. This stage is dominated by physical effects, with chemical reactions being unable to compete. During the 10–20 ps range, a transient balance is achieved between the physical bombardment effect and the consumption of ROS by chemical reactions. At this stage, ROS have penetrated to a certain depth within the DBP molecular layer, significantly increasing the contact area with DBP molecules and drastically raising the probability of reaction. This leads to the consumption of a large number of ROS by reactions before they can reach their potential maximum penetration depth, causing the growth of the average penetration depth to stagnate.

After passing the plateau phase (when ROS insertion stops), the average penetration depth decreases rapidly. The rate of longitudinal diffusion of ROS begins to fall below the consumption rate due to reactions in the surface region, causing the reaction front to recede toward the surface of the molecular layer. Simultaneously, ROS that have reacted to form small molecules gradually escape from the simulation system due to diffusion, macroscopically manifesting as a decrease in average penetration depth. Subsequently, as the reactant concentration decreases due to consumption, the simulation system gradually enters a stable reaction period. A large number of ROS that have participated in reactions and become small molecules gradually escape, and the kinetic energy of the ROS is largely depleted, preventing further penetration into deeper layers. This results in a slow increase in the average penetration depth.

The continuous increase in maximum penetration depth with time and kinetic energy demonstrates that, although the average penetration depth fluctuates and exhibits plateaus due to chemical reactions, an increase in kinetic energy enables some ROS to break through the barrier of reaction consumption and reach the deepest part of the contaminant molecular layer. This greatly increases the collision frequency between ROS and deep-layer DBP molecules, as well as the probability of chemical reactions occurring. Furthermore, this avoids the scenario where the reaction depth of ROS is too shallow, causing subsequently irradiated ROS to only further decompose surface reaction intermediates rather than decompose more DBP molecules. This further contributes to an increase in the DBP decomposition rate.

In summary, an increase in initial kinetic energy enhances the initial injection capability of ROS, rapidly expanding the early reaction interface. Although intense decomposition reaction consumption limits the increase in penetration depth, the strong penetration capability conferred by high kinetic energy ensures that ROS can react with deep-layer DBP molecules, preventing reaction stagnation due to diffusion limitations.

**Figure 8:**
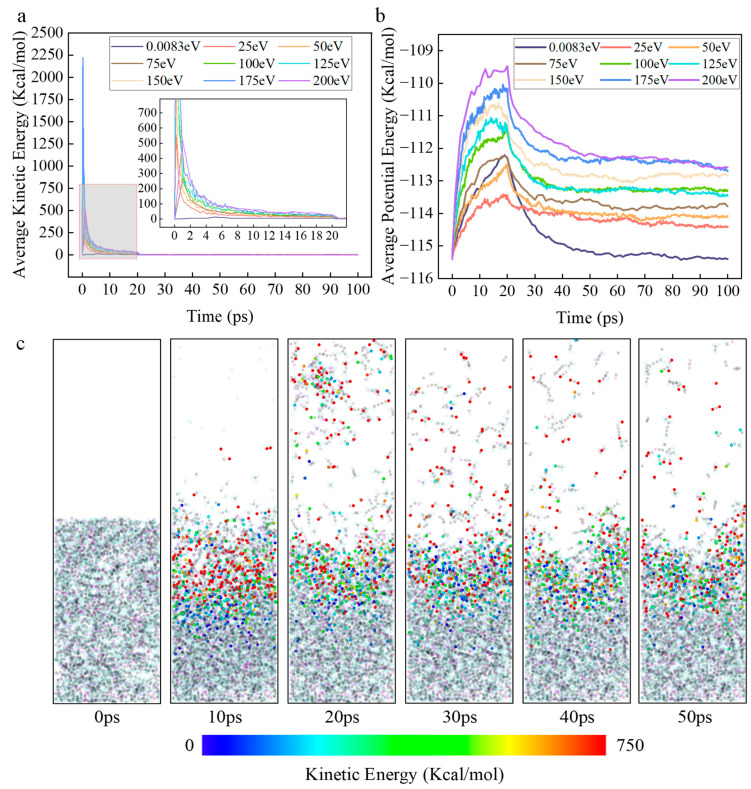
Schematic diagram of the conversion relationship between the kinetic energy of ROS and the potential energy of the system in the simulation. (**a**) Variation in the average kinetic energy of ROS with time and initial kinetic energy. (**b**) Variation in the system potential energy with time and initial kinetic energy. (**c**) Kinetic energy cloud map showing the temporal variation in ROS kinetic energy during the decomposition reaction simulation. In the figure, the semi-transparent black atoms are DBP carbon atoms, the semi-transparent blue atoms are DBP hydrogen atoms, the semi-transparent purple atoms are DBP oxygen atoms, and the opaque atoms are ROS particles indicating different kinetic energies.

Secondly, the variation in the average kinetic energy of ROS in the simulation system with time and initial kinetic energy, and the variation in the potential energy of the simulation system with time and initial kinetic energy, obtained through software processing, are shown in Figure 8a,b, respectively. Figure 8c displays the kinetic energy cloud map illustrating the change in kinetic energy of ROS particles over time during the simulated reaction process.

It was experimentally observed that the average kinetic energy of ROS in all groups with kinetic energy decreases sharply in the initial stage (0–20 ps) and eventually approaches zero, and the higher the initial kinetic energy, the slower the kinetic energy decay rate. This phenomenon clearly indicates that intense energy exchange and dissipation occur between the initial kinetic energy of ROS and the DBP system. The mechanism is that high-kinetic-energy ROS undergo inelastic collisions with DBP, converting their kinetic energy into two forms of energy: collisions cause vibration, stretching, and torsion of chemical bonds in DBP molecules, directly leading to an increase in the system’s potential energy, providing activation energy for chemical reactions; kinetic energy is dissipated by exciting molecular translation and rotation, equivalently raising the local instantaneous temperature of the simulation system.

Between 0–20 ps, the system potential energy increases uniformly and is positively correlated with the initial kinetic energy of ROS. This stage corresponds to the process of ROS being inserted stepwise and undergoing numerous collisions with DBP molecules. The higher the initial kinetic energy, the greater the total energy flux injected, so more energy is converted into potential energy within chemical bonds, exciting the simulation system to a higher energy barrier.

After 20 ps, the system potential energy of the zero-kinetic-energy group decreases more rapidly compared to the groups with kinetic energy and stabilizes at a lower steady-state value. This indicates that its energy release process is primarily completed through reactions after ROS diffusion with the surface of the DBP molecular layer, where the reaction area is limited, and the system quickly reaches energy balance. In contrast, after imparting initial kinetic energy, the system’s potential energy is maintained at a higher steady-state level. This phenomenon indicates that high initial kinetic energy endows ROS with high penetration capability, enabling reactions to occur not only on the surface. High kinetic energy results in an overall higher system potential energy level, meaning more DBP molecules are elevated above the reaction energy barrier. This directly leads to a faster reaction rate. Meanwhile, the slower the kinetic energy decay, the more sustained the energy supply, continuously driving decomposition and further increasing the decomposition rate. Secondly, the higher system of potential energy creates conditions for reaction pathways requiring higher energy activation. This is consistent with the previous analysis. For example, the ring-opening reaction of the benzene ring requires far more energy than the cleavage of the butyl side chain. Due to the low overall potential energy level, reactions in the low-kinetic-energy group preferentially occur along low-energy-barrier pathways. In contrast, high kinetic energy maintains a high potential energy environment, allowing high-energy-barrier pathways to occur and gradually become the dominant reaction pathways.

**Figure 9:**
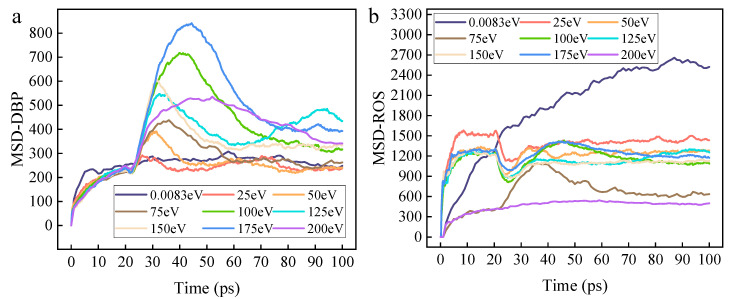
Schematic diagram of the MSD changes in particles in the system. (**a**) Graph of the relationship between the average MSD of the DBP example and the initial kinetic energy over time. (**b**) Graph of the MSD of ROS varying with time and initial kinetic energy.

Subsequently, the variation in the mean MSD of DBP molecules and the MSD of ROS with time and kinetic energy, calculated based on atomic coordinates, is shown in Figure 9a,b, respectively. Under all incident kinetic energies, the MSD of DBP rises rapidly within 0–10 ps, exhibiting a significant transient peak at 30–45 ps. This peak shows a non-monotonic variation with kinetic energy: medium kinetic energies (approximately 100–125 eV) produce the highest overshoot, while lower (50–75 eV) and higher (150–200 eV) kinetic energies yield smaller peaks. After the peak, the MSD gradually decreases over approximately 20–40 ps, stabilizing for most cases by 80–100 ps. The mobility of ROS is significantly higher than that of DBP. Its MSD climbs rapidly within 0–10 ps. Multiple curves show an early plateau at 10–20 ps, followed by a decline and stabilization between 40–60 ps. As kinetic energy increases, the initial slope and steady-state level generally rise. The positive correlation between MSD of ROS and kinetic energy indicates enhanced transport that assists chemical reactions by increasing encounter frequency and penetration; however, the elementary removal steps remain chemical in nature. High-kinetic-energy ROS significantly enhance the effective collision frequency with DBP molecules through increased mobility (higher MSD slope and steady-state value), which is the primary reason for the macroscopic reaction rate increase with kinetic energy. Furthermore, the data indicate that medium kinetic energies (~100–125 eV) most effectively excite transient large-scale structural reorganization in DBP molecules, a phenomenon closely related to reaction pathway selection. This energy range most efficiently converts the kinetic energy of ROS into energetic excitation of DBP molecules, whereas excessively high kinetic energy may lead to over-penetration, dissipating energy before it is fully transferred to DBP; insufficient kinetic energy fails to effectively excite DBP motion. The mobility of ROS is markedly higher than that of DBP (MSD is 2–5 times greater), and this gap further widens at higher kinetic energies. This implies that ROS remains the driver of the reaction. This asymmetry in mobility allows the reaction front to advance rapidly into the material interior, while DBP fragments remain relatively localized. This explains why the reaction removal rate is higher under high kinetic energy conditions.

## 3. Simulation Model

Based on the ReaxFF reactive force field, molecular dynamics simulations utilize bond-order as the core concept, enabling the spontaneous formation and breaking of bonds within atomic trajectories to simulate the chemical reactions between reactive oxygen species from plasma and organic contaminants [38,39]. Simultaneously, this simulation method allows for the application of initial velocities to the incident reactive oxygen species, facilitating the individual investigation of the effects of initial kinetic energy, flux of reactive oxygen species, irradiation dose, and ambient temperature on cleaning efficiency and reaction pathways by adjusting simulation system parameters. In summary, since ReaxFF-based molecular dynamics simulations can simulate both kinetically assisted chemical removal and chemistry-only control, enabling a clean separation of kinetic promotion and intrinsic chemistry, thereby enabling the decoupling of physical and chemical effects, this method is selected for simulating the plasma-driven removal reactions of organic contaminants [40].

**Figure 10:**
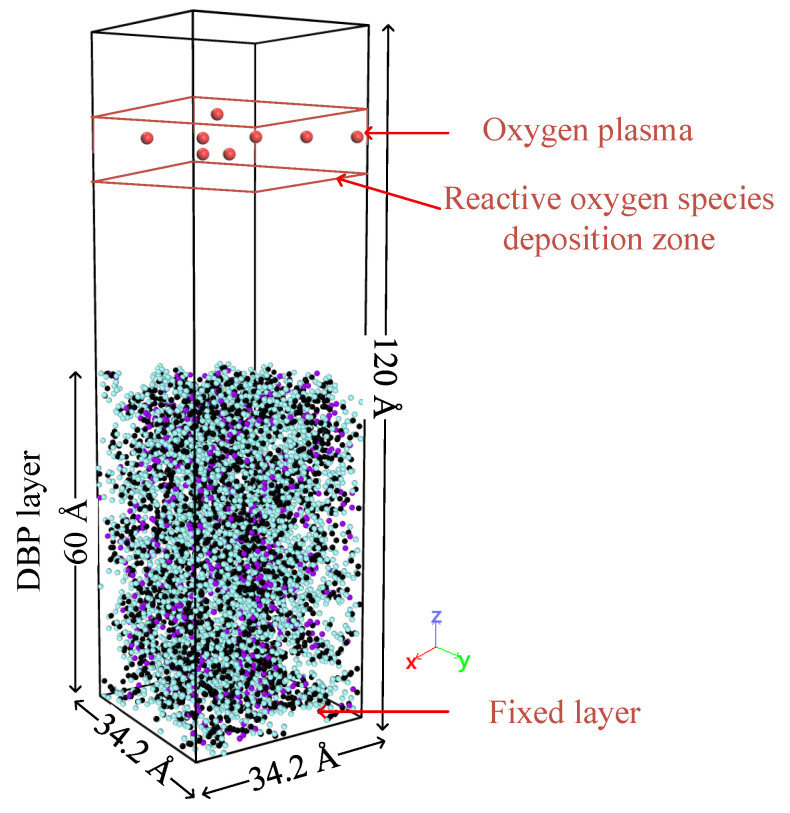
Schematic diagram of simulation system construction. In the figure, the black spheres represent the carbon atoms of DBP, the cyan spheres represent the hydrogen atoms of DBP, the purple spheres represent the oxygen atoms of DBP, and the red spheres represent the active oxygen groups.

Although real oxygen plasmas contain ions, electrons, and metastable species, the model selected neutral O atoms as representative ROS due to their high abundance and reactivity in non-thermal oxygen plasmas. The simulation model does not include charged species or self-consistent fields; thus, ion-induced sputtering lies outside the present scope. The assigned translational kinetic energy of incident O models sheath-accelerated impacts to isolate how kinetic factors promote ROS–DBP chemistry reaction. The selection of atomic oxygen as the primary bombarding species in our simulations was also informed by Optical Emission Spectroscopy (OES) measurements from our experiments, which identified O as a dominant reactive species in our low-pressure air plasma. Based on previous research findings on the composition of surface contaminants on optical components, dibutyl phthalate (DBP) was selected as a representative organic contaminant. This choice is justified by gas chromatography–mass spectrometry (GC-MS) analyses of actual contaminants within laser facilities, which identified DBP as a prevalent component [17]. DBP is highly volatile in vacuum environments and adheres to optical component surfaces upon contact, containing a benzene ring, ester group, and carbon chains, enabling it to represent the chemical properties of most organic contaminants (alkanes, aromatic hydrocarbons, esters) found in intense laser facilities. Initially, a DBP molecular cluster with a density of 1.053 g/cm^3^ (consistent with the density of DBP at room temperature) was constructed, followed by geometric optimization and energy minimization. Subsequently, a simulation box measuring 34.2 Å × 34.2 Å × 120 Å was constructed, into which the DBP molecular cluster was imported. Periodic boundary conditions were applied in the X and Y directions to simulate an infinite boundary system, while the Z direction was set as a fixed reflective boundary to prevent reactive oxygen species from penetrating and escaping beyond the organic contaminant molecular layer. Energy minimization was then performed, and the organic contaminant molecular layer was relaxed to the system-set temperature under the NVT (N—particle number, V—volume, T—temperature) ensemble. After system stabilization, the organic contaminant molecular layer measured 34.2 Å × 34.2 Å × 60 Å, with its bottom layer fixed at a thickness of 5 Å to prevent substrate movement during simulation from affecting the calculation results. A vacuum layer was then added above the DBP molecular layer, followed by the generation of reactive oxygen species with initial velocities at random positions within a fixed height region of 95 Å–105 Å. The system simulation time step was set to 0.2 fs to enhance computational accuracy. The schematic diagram of the simulation system is shown in Figure 10. Detailed parameters and environmental conditions for the molecular dynamics simulations, along with selected simulation data, are provided in the Supplementary Materials. The data of instantaneous abundance of multiple reaction products and weights of different reaction pathways can be seen in Tables S1 and S2; The data on the variation of the average penetration depth and the maximum penetration depth of ROS are provided in Tables S3 and S4.

Since plasma cleaning of organic contaminants is primarily influenced by plasma energy, flux, and ambient temperature, the main experimental variables selected for simulating reactive oxygen species are initial kinetic energy, irradiation flux, irradiation dose, and ambient temperature [41]. These experimental variables are derived from system setting parameters in the simulation, such as the initial velocity of oxygen species, the time interval for inserting oxygen species, the number of oxygen atoms inserted, and the ensemble temperature. To observe significant interaction effects on the picosecond timescale, the simulation experiments set a wide range of parameters for oxygen plasma, an approach widely used in other molecular dynamics simulation studies. Based on preliminary experimental results, the initial kinetic energy of 75 eV, irradiation flux of 2.137 × 10^26^ cm^−2^s^−1^, irradiation dose of 500, and ambient temperature of 300 K (room temperature) were selected as standard experimental parameters. Subsequently, single-factor experiments were conducted, with parameters not being tested set according to the standard experimental parameters.

In the simulations, the incident velocities of reactive oxygen species were set to 0.01 Å/fs, 0.17 Å/fs, 0.25 Å/fs, 0.30 Å/fs, 0.35 Å/fs, 0.39 Å/fs, 0.43 Å/fs, 0.46 Å/fs, and 0.50 Å/fs, corresponding to initial kinetic energies of 0.0083 eV, 25 eV, 50 eV, 75 eV, 100 eV, 125 eV, 150 eV, 175 eV, and 200 eV, respectively. The particle kinetic energy was calculated using the kinetic energy formula. To quantitatively analyze the contribution of physical effects to the cleaning reaction, a control group with an initial kinetic energy of 0.0083 eV was specifically established. Since this energy is much lower than the initial kinetic energy under normal parameter settings, it can be approximately considered that the reactive oxygen species in this group possess no initial kinetic energy, and the cleaning relies on chemistry without added kinetic assistance (no initial translational energy).

In the simulation, reactive oxygen species were inserted at intervals of 10 fs, 20 fs, 30 fs, 40 fs, 50 fs, 60 fs, 70 fs, and 80 fs, corresponding to irradiation fluxes of 8.547 × 10^26^ cm^−2^s^−1^, 4.274 × 10^26^ cm^−2^s^−1^, 2.849 × 10^26^ cm^−2^s^−1^, 2.137 × 10^26^ cm^−2^s^−1^, 1.709 × 10^26^ cm^−2^s^−1^, 1.425 × 10^26^ cm^−2^s^−1^, 1.221 × 10^26^ cm^−2^s^−1^, and 1.068 × 10^26^ cm^−2^s^−1^, respectively. The number of inserted reactive oxygen species served as the plasma irradiation dose, set to 200, 300, 400, 500, 600, 700, 800, and 900. To fully observe the complete interaction process between the plasma and the organic contaminant layer and allow the system to reach a steady state through complete reaction, the simulation continued for a period after the insertion of reactive oxygen species was completed. Changes in irradiation flux and irradiation dose affect the total insertion time of reactive oxygen species. Therefore, to ensure consistent experimental duration, the system relaxation time after insertion was fixed at 80 ps, eliminating the influence of varying insertion times on experimental comparisons.

Due to potential continuous temperature increases during cleaning, such as from physical collisions and exothermic chemical reactions, the Berendsen thermostat method was used to control temperature during the simulation process. The Berendsen thermostat was also employed to maintain the ambient temperature at the experimentally set values. The Berendsen thermostat method is a weak-coupling approach that simulates heat exchange between the system and the external environment, enabling the system to quickly reach the set temperature while minimizing interference with the calculation results. During the simulation, the system temperature was set to 225 K, 250 K, 275 K, 300 K, 325 K, 350 K, 375 K, and 400 K, respectively.

## 4. Conclusions

The study results show that chemical reactions between ROS and DBP are primary, whereas initial kinetic energy promotes efficiency and pathway access by enhancing transport, penetration, and local energy transfer. The cooperative effects of flux, irradiation dose and ambient temperature further assist the chemical reaction, offering atomistic guidance for kinetically assisted optimization of plasma cleaning. The ReaxFF MD simulation methodology proves powerful in decoupling complex physical–chemical interactions, providing atomic-level insights into transient reaction processes, energy exchange, and pathway evolution that are inaccessible to experimental techniques. The core conclusions are as follows. The initial kinetic energy of ROS is a key kinetic promoter that modulates efficiency and pathways within a chemically driven mechanism. It dramatically enhances the cleaning reaction, far exceeding the effects of pure chemical reactions alone, by increasing the decomposition rate by over an order of magnitude and significantly reducing the final residue ratio. The promotion mechanism primarily operates through three interconnected aspects: enhanced diffusion and transport (higher MSD, greater penetration depth), direct energy transfer and excitation (elevating system potential energy, providing activation energy for bond breaking), and selective activation of reaction pathways. Kinetic energy allows ROS to overcome specific energy barriers, sequentially promoting mono-butyl cleavage, dual-butyl cleavage, and ultimately direct benzene ring cleavage as the energy increases. Synergistic effects exist between kinetic energy and other parameters. An optimal irradiation flux exists for maximum efficiency, beyond which surface passivation may occur. The irradiation dose ensures the thoroughness of the reaction initiated by kinetic energy. Ambient temperature provides additional thermal driving force, coupling with kinetic energy to lower effective reaction barriers and promote product desorption.

In summary, this work establishes that chemistry is the core removal mechanism, while kinetic energy is a strong promoter that enhances transport and activation to achieve efficient plasma cleaning. It provides a comprehensive theoretical framework and quantitative basis for precisely optimizing plasma parameters (especially kinetic energy and flux) in practical applications to maximize cleaning efficiency while minimizing substrate damage and energy consumption. Future work could explore a wider range of contaminant molecules and the interplay between different plasma species.

## Data Availability

The raw data supporting the conclusions of this article will be made available by the authors on request.

## Supplementary Materials

The following supporting information can be downloaded at: https://www.mdpi.com/article/10.3390/molecules30194010/s1, Table S1: The data of instantaneous abundance of multiple reaction product varying with kinetic energy; Table S2: Data on the variation of weights of multiple reaction routes with kinetic energy; Table S3: Data on the variation of the average penetration depth of ROS with time and initial kinetic energy under standard conditions; Table S4: Data on the variation of the maximum penetration depth of ROS with time and initial kinetic energy under standard conditions.
