# Supplementary material for: Kinetically Assisted Chemical Removal of Organic Contaminants by Reactive Oxygen Species: Insights from ReaxFF Molecular Dynamics Simulations
Source: Molecules. 2025 Oct 7;30(19):4010. doi: 10.3390/molecules30194010 (PMC12526405; doi:10.3390/molecules30194010)
Supplement: Supplementary file 1 [file molecules-30-04010-s001.zip › molecules-3876294-supplementary.pdf]

molecules

The Supplementary Materials

Kinetically Assisted Chemical Removal of Organic Contaminants by Reactive Oxygen Species: Insights from ReaxFF Molecular Dynamics Simulations

Zixu Wang [1], Yuhai Li [2,*], Peng Zhang [3,4,*], Fei Wang [2,3], Laixi Sun [2], Qingshun Bai [3], Mingzhi Zhu [1,*] and Baoxu Wang [1]

1 Institute of Systems Engineering, China Academy of Engineering Physics, Mianyang 621999, China
2 Laser Fusion Research Center, China Academy of Engineering Physics, Mianyang 621900, China
3 School of Mechatronics Engineering, Harbin Institute of Technology, Harbin 150000, China
4 Chongqing Research Institute, Harbin Institute of Technology, Chongqing 401135, China
* Correspondence: 15546027805@163.com (Y. Li), zp@hit.edu.cn (P. Zhang) and zhumz@caep.cn (M. Zhu)

Academic Editor: Adam Liwo

Received: 31 August 2025
Revised: 21 September 2025
Accepted: 2 October 2025
Published: 7 October 2025

Citation: Wang, Z.; Li, Y.; Zhang, P.; Wang, F.; Sun, L.; Bai, Q.; Zhu, M.; Wang, B. Kinetically Assisted Chemical Removal of Organic Contaminants by Reactive Oxygen Species: Insights from ReaxFF Molecular Dynamics Simulations. Molecules **2025**, 30, 4010. https://doi.org/10.3390/molecules30194010

Copyright: © 2025 by the authors. Submitted for possible open access publication under the terms and conditions of the Creative Commons Attribution (CC BY) license (https://creativecommons.org/licenses/by/4.0/).

The Minimum Data Set includes information such as the runnable code for reactive molecular dynamics simulations, environmental configuration, basic information settings, and boundary condition settings. It also comprises the data results from the simulations, including the DBP residue ratio and average penetration depth under specific experimental conditions.

Runnable code for reactive molecular dynamics simulations

mental conditions.

```
dimension        3

units            real            # mass = grams/mole, distance = Angstroms, time = femtoseconds, energy = Kcal/mole, velocity = Angstroms/femtosecond, temperature = Kelvin

boundary         p p f           # p = periodic, f= fixed

atom_style       charge          # Classical particles

read_data 5-160DBP.data

variable num_tep   equal 300.0

variable num_vol   equal 0.3

variable num_mol   equal 500.0

variable num_flux equal 200.0

variable num_step equal 500000

change_box       all z delta 0 60 units box
```

```
region                    sput block 0 33 0 32 95 105

region   bot block INF INF INF INF INF 5 units box

group fbot region bot

group DBP type 1 2 3 4 5 6 7

group O_plasma type 8

pair_style reaxff   NULL

pair_coeff * * ffield.reax.cho C C O O C C H O #baffield.reax.CHO;ffield.reax.cho

fix qeq all qeq/reaxff 1 0.0 10.0 1.0e-8 reaxff

neighbor      2.0 bin

neigh_modify delay 0 every 10 check no

timestep   0.2

comm_style   tiled

balance       1.1 rcb

thermo        1000

thermo_modify lost ignore flush yes

fix   setf fbot setforce 0.0 0.0 0.0

min_style    cg

minimize      1e-10 1e-10 5000 5000

reset_timestep 0

fix 1 DBP nvt temp ${num_tep} ${num_tep} 20.0

fix tember DBP temp/berendsen ${num_tep} ${num_tep} 20.0

fix temNVE O_plasma nve

fix dep O_plasma deposit ${num_mol} 8 ${num_flux} 95485 region sput vz -${num_vol} -
${num_vol}

fix zwal all wall/reflect zlo EDGE

compute ke O_plasma ke/atom

compute pe all pe/atom

compute msd all msd com yes
```

```
variable msdx equal c_msd[1]

variable msdy equal c_msd[2]

variable msdz equal c_msd[3]

variable msd    equal c_msd[4]

variable istep equal step

fix msd all print 500 "${istep} ${msdx} ${msdy} ${msdz} ${msd}" screen no file msd.dat

dump 1 all custom 500 T${num_tep}-V${num_vol}-M${num_mol}-F${num_flux}-S${num_step}.lammpstrj id type x y z c_ke c_pe

dump 2 all custom 5000 T${num_tep}-V${num_vol}-M${num_mol}-F${num_flux}-S${num_step}.*.lammpstrj id type x y z c_ke c_pe

dump_modify 1 sort id

fix speout all reaxff/species 1 1 500 species.out element C C O O C C H O
run ${num_step}
```

The variation in instantaneous abundance of characteristic products with initial kinetic energy under the conditions of 5 ps, an irradiation flux of 2.137×10^{26} $cm^{-2}s^{-1}$, an irradiation dose of 500, and an ambient temperature of 300 K.
Table S1: The data of instantaneous abundance of multiple reaction product varying with kinetic energy.

Initial Kinetic Energy -5ps	Instantaneous Abundance	Instantaneous Abundance	Instantaneous Abundance	Instantaneous Abundance
eV				*10
	n(C_6)	n(C_8)	n(C_{12})	Small-molecule Product (*10)
0.0083	0.50625	0.24063	1.4875	0.64063
25	0.6625	0.68125	1.8875	1.41
50	0.89375	0.89375	1.45625	1.81875
75	0.7375	1.425	1.39375	1.97375
100	1.00625	1.36875	1.3875	2.51813
125	0.9625	1.5125	1.26875	2.64938
150	1.38125	1.7875	1.2125	2.85063
175	1.625	1.48125	1.06875	2.95188
200	1.10938	1.49688	0.95	3.30938

The variation in Reaction Weight of different reaction pathways with initial kinetic energy under the conditions of 5 ps, an irradiation flux of 2.137×10^{26} cm^{-2}s^{-1}, an irradiation dose of 500, and an ambient temperature of 300 K.

Table S2: Data on the variation of weights of multiple reaction routes with kinetic energy.

Initial Kinetic Energy -5ps	Reaction Weight	Reaction Weight	Reaction Weight
eV	%		
	R_{Ph}	R_{DB}	R_{SB}
0.0083	22.65734	10.76923	66.57343
25	20.5029	21.08317	58.41393
50	27.55299	27.55299	44.89403
75	20.73814	40.0703	39.19156
100	26.74419	36.37874	36.87708
125	25.70952	40.40067	33.88982
150	31.52639	40.79886	27.67475
175	38.92216	35.47904	25.5988
200	31.19508	42.09139	26.71353

The variation in average penetration depth and maximum penetration depth of reactive oxygen species (ROS) particles with initial kinetic energy under the conditions of 5 ps, an irradiation flux of 2.137×10^{26} cm^{-2}s^{-1}, an irradiation dose of 500, and an ambient temperature of 300 K.

Table S3: Data on the variation of the average penetration depth of ROS with time and initial kinetic energy under standard conditions.

Time	Average penetration depth												
ps	Kcal/mol												
	0.0083 eV		25eV	50eV		75eV		100eV	125eV	150eV	175eV		200eV
0	0	0	0	0	0	0	0	0	0	0	0	0	0
1	2.37989	0.2	552.7272	823.37158	1	254.86138	0.2	1437.7005	1496.73726	1950.35858	2218.8906	1	518.96113
2	5.15735	0.4	362.01692	444.58616	2	128.03132	0.4	818.93619	863.54769	998.15187	997.11978	2	259.72429
3	6.27503	0.6	220.31724	334.04445	3	91.3203	0.6	648.36474	575.96029	886.00874	899.20426	3	158.38132

4	5.01925	0.8	165.11629	294.0202	4	63.46122	0.8	508.66378	470.81579	601.57226	696.31408	4	135.07323
5	12.82978	1	134.02464	201.51043	5	55.67085	1	250.59853	441.70692	417.38475	446.0253	5	92.83852
6	11.40336	1.2	111.77748	174.57111	6	45.41581	1.2	277.44542	220.42232	401.4208	314.19435	6	87.35859
7	6.86123	1.4	105.13118	163.3968	7	36.80291	1.4	209.55055	249.08451	266.27107	286.09998	7	82.09238
8	6.54219	1.6	91.78457	123.41235	8	44.11534	1.6	177.58923	194.44012	260.55666	296.27377	8	69.43015
9	6.73024	1.8	87.45089	126.85404	9	37.8911	1.8	193.71579	197.04051	216.86231	248.35619	9	66.59448
10	5.87351	2	76.80165	106.34053	10	33.69525	2	149.90867	167.64581	219.24652	216.68008	10	60.06479
11	7.24932	2.2	70.90578	106.44087	11	32.82213	2.2	141.98362	191.66164	223.4075	254.9822	11	60.36154
12	6.6009	2.4	68.67911	108.96093	12	28.6558	2.4	120.74178	145.89686	203.92041	189.70962	12	44.10303
13	6.67068	2.6	59.92914	94.23248	13	23.79356	2.6	135.47608	150.54043	186.84445	136.48496	13	46.16571
14	6.41252	2.8	56.46576	78.4969	14	20.38159	2.8	113.08441	160.50853	175.48302	189.49825	14	40.7522
15	5.55444	3	53.0681	76.31578	15	22.66169	3	113.73004	119.14157	148.72126	151.24777	15	49.64325
16	5.74508	3.2	40.94612	73.88884	16	19.69741	3.2	125.66289	126.91614	143.8224	147.09032	16	42.79704
17	5.87015	3.4	43.8175	75.50999	17	20.28319	3.4	106.90856	145.59711	141.50892	142.53237	17	40.04744
18	6.37069	3.6	54.67093	69.89827	18	21.81611	3.6	117.66	122.16757	146.60968	154.52727	18	38.90262
19	6.18903	3.8	46.03586	69.03162	19	17.82752	3.8	84.64599	103.37221	149.31341	153.05964	19	30.56275
20	5.73008	4	36.33785	56.46734	20	17.4631	4	87.06904	79.91549	122.03363	113.91596	20	28.45307
21	5.84322	4.2	37.42523	58.13299	21	2.56029	4.2	87.88977	107.99979	92.21799	99.17178	21	1.28505
22	6.41132	4.4	41.44629	50.74686	22	2.34612	4.4	84.95902	87.29464	95.4154	101.96793	22	1.1324
23	5.49308	4.6	31.30978	54.5604	23	2.22886	4.6	75.61398	86.23979	95.07594	121.21184	23	1.12452
24	6.20421	4.8	38.18094	46.52018	24	2.22181	4.8	68.3136	80.77399	95.96525	90.96162	24	1.03229

25	5.89484	5	33.88664	42.33358	25	1.98933	5	75.74305	80.53528	92.66334	104.47335	25	0.96289
26	5.51669	5.2	37.14855	50.01723	26	2.03581	5.2	74.56111	65.09656	97.53024	106.56633	26	0.97472
27	5.21588	5.4	35.17082	47.64756	27	1.89528	5.4	75.88583	67.95691	83.62833	83.70851	27	0.99709
28	4.97237	5.6	26.41757	47.91819	28	2.0951	5.6	75.54669	69.01689	76.09828	86.64629	28	0.99831
29	4.57129	5.8	31.14249	45.59418	29	1.96561	5.8	63.44777	71.39581	94.13829	81.85469	29	0.90421
30	4.57382	6	28.04411	38.85974	30	2.22215	6	58.69199	53.18661	67.98373	77.8331	30	0.90456
31	4.61207	6.2	29.04041	46.13771	31	2.01972	6.2	66.14331	52.94292	78.5672	79.05792	31	0.90605
32	4.56891	6.4	29.22417	47.35788	32	1.77906	6.4	57.78695	66.01602	86.94746	102.41542	32	0.91165
33	4.58738	6.6	25.62835	35.8662	33	2.12427	6.6	53.69115	64.46925	81.3721	76.69782	33	0.94382
34	5.31073	6.8	23.06318	47.23356	34	2.01988	6.8	75.83691	61.71097	70.91007	78.96168	34	0.89396
35	5.07716	7	30.58956	38.79777	35	2.08084	7	56.23203	65.22021	78.33441	84.59364	35	0.90343
36	5.74649	7.2	24.61211	37.2894	36	2.07839	7.2	56.40584	62.07637	61.7537	69.05465	36	0.90585
37	5.38744	7.4	26.65043	34.65758	37	1.8945	7.4	57.79324	68.58023	68.49896	77.32319	37	0.91817
38	4.24237	7.6	23.68077	34.40851	38	1.88908	7.6	53.56382	61.79232	59.72145	69.67711	38	0.90215
39	4.08964	7.8	26.69723	35.02956	39	2.07633	7.8	45.81822	51.63378	61.62249	70.15062	39	0.90011
40	4.24155	8	23.36546	30.20777	40	2.01664	8	44.98024	57.20246	54.11436	57.93364	40	0.83811
41	3.90641	8.2	25.28857	30.97628	41	1.97608	8.2	43.37748	45.64294	61.98856	60.75594	41	0.89524
42	4.06861	8.4	26.11457	32.1388	42	2.15761	8.4	43.18128	43.23293	47.2267	55.82367	42	0.88166
43	4.22687	8.6	24.50364	29.23136	43	2.13766	8.6	48.56134	54.85812	57.01092	53.43506	43	0.77332
44	4.00856	8.8	22.4316	31.58326	44	1.88821	8.8	42.34816	45.95892	57.37891	57.53019	44	0.90031
45	4.0661	9	23.88666	32.02337	45	1.95136	9	41.29647	45.7622	51.45585	59.85374	45	0.81007

46	4.12034	9.2	22.30518	25.64379	46	1.80108	9.2	41.57629	48.91611	55.06811	53.61102	46	0.87361
47	3.648	9.4	20.33782	29.17026	47	1.89649	9.4	32.25086	35.35755	48.06435	45.81089	47	0.83969
48	3.36128	9.6	19.94176	29.26732	48	1.89508	9.6	38.35593	44.61634	49.91175	53.0135	48	0.79145
49	3.94966	9.8	20.13797	24.63406	49	1.89417	9.8	34.59539	38.61821	46.26603	42.69969	49	0.8082
50	3.55351	10	20.30738	28.66235	50	2.02885	10	39.29448	42.16092	54.70748	51.95826	50	0.81077
51	3.09615	10.2	22.60382	25.0695	51	1.89052	10.2	31.98485	36.81333	36.66581	54.81635	51	0.8884
52	3.3365	10.4	18.14256	26.44585	52	1.89903	10.4	38.19946	36.04026	45.69233	54.01146	52	0.80303
53	3.88745	10.6	18.91871	26.23323	53	1.93909	10.6	36.86086	39.95054	44.72916	51.62788	53	0.83095
54	3.73676	10.8	16.60178	27.00429	54	2.0032	10.8	37.19778	38.09879	44.41014	45.98198	54	0.80373
55	3.79617	11	19.34136	30.20765	55	2.17135	11	34.64758	45.0075	48.06341	46.22666	55	0.82542
56	3.66468	11.2	19.43346	25.6945	56	2.28489	11.2	37.62287	33.6795	50.71855	43.80303	56	0.78673
57	3.35312	11.4	21.00202	22.4915	57	2.19995	11.4	29.3053	40.95248	42.5732	45.32917	57	0.77945
58	2.82969	11.6	19.83231	21.92817	58	1.9478	11.6	28.40631	41.6096	39.97419	46.29141	58	0.79858
59	3.39033	11.8	18.6352	24.15419	59	2.00009	11.8	35.91975	29.002	43.97462	45.60945	59	0.88657
60	3.10335	12	17.15198	23.61792	60	1.88462	12	31.96123	37.01628	41.04642	45.97649	60	0.76835
61	3.15483	12.2	16.90562	21.18504	61	1.92141	12.2	27.59853	35.79153	28.87457	42.61337	61	0.83544
62	2.83681	12.4	16.7888	22.8706	62	1.8262	12.4	32.41386	36.30774	40.6464	46.5067	62	0.8001
63	3.05944	12.6	18.42402	21.46104	63	1.87224	12.6	29.57883	36.98442	37.25348	39.30945	63	0.85074
64	3.02657	12.8	17.46437	22.79593	64	2.13202	12.8	27.94094	29.76895	33.97391	37.74282	64	0.88229
65	3.20881	13	17.53049	23.31473	65	2.01273	13	25.28858	29.69704	43.00122	38.01897	65	0.83342
66	2.68179	13.2	18.44331	22.53512	66	1.76813	13.2	25.98463	34.07728	40.01307	37.3125	66	0.84484

67	2.88874	13.4	18.52433	22.71391	67	2.06546	13.4	22.56011	34.154	38.70306	38.05164	67	0.80547
68	2.60351	13.6	17.23054	19.67461	68	1.95255	13.6	25.88024	30.19677	30.12532	38.92595	68	0.81802
69	2.58426	13.8	17.56238	17.50831	69	1.77869	13.8	25.90093	28.27947	36.84641	40.54452	69	0.81441
70	3.12414	14	15.43616	19.19056	70	1.81273	14	24.07292	33.2815	37.62616	33.991	70	0.82307
71	2.47832	14.2	15.1028	21.79568	71	1.90276	14.2	26.80702	33.10678	30.93693	43.48382	71	0.82294
72	2.58642	14.4	16.22527	19.37982	72	1.8743	14.4	24.79732	29.66195	32.10102	39.45975	72	0.79163
73	2.31541	14.6	17.43139	22.98255	73	1.69117	14.6	26.4629	26.4108	33.14927	37.30886	73	0.74582
74	2.61838	14.8	18.02949	21.1205	74	1.87074	14.8	29.59411	29.68865	33.21854	40.85189	74	0.74906
75	2.30539	15	14.51775	20.66915	75	1.98985	15	28.98299	32.45374	37.32057	42.93527	75	0.79816
76	2.16269	15.2	14.5371	22.43972	76	1.74659	15.2	27.51223	30.00182	33.23298	29.929	76	0.78194
77	2.37955	15.4	15.51857	18.28211	77	2.01321	15.4	23.42941	29.55212	30.73752	33.21415	77	0.80101
78	2.56272	15.6	16.71013	18.49939	78	1.82254	15.6	27.09455	26.52327	30.62459	32.43918	78	0.8518
79	2.22113	15.8	13.87483	21.19799	79	1.8752	15.8	29.10725	31.00412	40.01608	39.33572	79	0.77391
80	2.17715	16	16.144	17.07404	80	1.74401	16	20.99138	28.9742	30.62036	33.33387	80	0.78522
81	2.12287	16.2	16.11705	17.74345	81	1.8735	16.2	24.68487	25.98781	28.45985	30.10149	81	0.77006
82	2.45395	16.4	14.18675	19.28189	82	1.76111	16.4	20.51256	23.5493	26.13898	30.44491	82	0.80015
83	2.41635	16.6	13.12834	19.62953	83	1.91804	16.6	23.69178	28.859	30.78243	31.86875	83	0.8137
84	2.18951	16.8	13.5766	18.9584	84	1.63889	16.8	26.37407	26.69233	25.06543	33.76933	84	0.82077
85	2.24763	17	14.54267	18.08413	85	1.77982	17	23.94004	28.45617	27.50859	35.51303	85	0.77724
86	2.1233	17.2	13.99942	17.45933	86	1.9505	17.2	22.36784	26.41869	27.80302	31.15675	86	0.82602
87	2.10467	17.4	13.29713	17.20261	87	1.84951	17.4	22.90925	20.27874	28.66537	24.57564	87	0.80557

88	1.91975	17.6	12.1116	17.16434	88	1.9142	17.6	24.54725	20.36617	29.7928	26.16938	88	0.81504
89	2.18431	17.8	13.51344	17.47468	89	1.88526	17.8	19.37185	25.46696	29.3311	28.29455	89	0.84588
90	1.84334	18	15.2903	18.04761	90	1.87785	18	24.16541	23.55071	27.86078	30.18068	90	0.78485
91	2.15342	18.2	14.30865	19.85027	91	1.99955	18.2	22.37881	25.66846	30.62346	29.66771	91	0.83736
92	2.043	18.4	14.17187	16.28784	92	1.96567	18.4	18.59686	26.04402	24.43776	31.23576	92	0.76774
93	1.86399	18.6	12.82967	18.02003	93	2.0277	18.6	22.29428	24.89551	27.41817	26.88244	93	0.76865
94	2.01043	18.8	13.4464	17.17948	94	1.86009	18.8	22.23622	22.47852	26.6346	28.36827	94	0.81512
95	2.11578	19	12.56941	15.14839	95	1.94985	19	20.45817	25.398	30.03457	24.69094	95	0.81792
96	2.23847	19.2	12.92872	16.15471	96	1.7931	19.2	23.71269	21.56286	21.76478	23.67068	96	0.77809
97	2.10478	19.4	12.99029	17.10813	97	1.77522	19.4	18.90399	21.50985	24.72613	28.9724	97	0.76944
98	2.50604	19.6	12.87248	15.99592	98	1.77409	19.6	21.04063	23.7665	22.76893	25.58922	98	0.78817
99	2.38886	19.8	13.46527	15.36319	99	1.84164	19.8	19.59906	21.45565	24.74707	25.83807	99	0.79066
100	2.07898	20	12.48486	15.57722	100	1.77868	20	20.64423	22.52653	26.4701	25.39	100	0.82314

Table S4: Data on the variation of the maximum penetration depth of ROS with time and initial kinetic energy under standard conditions.

Time	Maximum penetration depth												
ps	Kcal/mol												
	0.0083eV		25eV	50eV		75eV		100eV	125eV	150eV	175eV		200eV
0	0	0	0	0	0	0	0	0	0	0	0	0	0
1	-26.6465	0.2	-4.274	9.4172	1	38.5338	0.2	17.1941	18.1968	20.8028	32.3914	1	518.96113
2	-14.2935	0.4	10.8075	17.5483	2	41.8801	0.4	23.134	28.2686	32.5218	39.2196	2	259.72429

3	9.3176	0.6	15.7373	24.5332	3	42.6882	0.6	28.7972	32.1887	40.1173	45.6399	3	158.38132
4	4.0154	0.8	17.8576	26.3669	4	43.7265	0.8	38.6912	33.6768	51.20254	45.1775	4	135.07323
5	10.6426	1	17.8955	27.0462	5	44.0445	1	39.9492	35.2506	50.5587	46.1048	5	92.83852
6	13.1275	1.2	18.0298	26.0422	6	44.5431	1.2	39.3686	40.3228	48.9841	46.6984	6	87.35859
7	13.8926	1.4	18.8105	27.8841	7	44.4878	1.4	40.3648	40.234	48.864	46.539	7	82.09238
8	16.5666	1.6	21.6818	27.2516	8	44.7375	1.6	41.805	41.6257	49.3233	46.3082	8	69.43015
9	16.1907	1.8	22.1038	28.0292	9	44.9898	1.8	41.53	41.2749	49.9104	53.16333	9	66.59448
10	16.4897	2	22.9605	28.781	10	45.0324	2	41.5785	40.3621	49.9301	59.11831	10	60.06479
11	21.915	2.2	23.1474	28.8789	11	45.0519	2.2	41.5848	40.2512	54.9143	51.18152	11	60.36154
12	22.6135	2.4	22.787	29.8043	12	45.0767	2.4	41.8663	40.3025	51.9244	50.87023	12	44.10303
13	23.0744	2.6	23.6137	30.8834	13	44.906	2.6	42.0513	39.8518	52.62951	52.17437	13	46.16571
14	22.5576	2.8	23.7748	33.3031	14	44.938	2.8	42.1247	45.2361	52.82678	53.15648	14	40.7522
15	23.1325	3	24.7639	33.5956	15	44.8529	3	42.2712	44.6774	53.04388	53.89452	15	49.64325
16	25.2873	3.2	24.8094	32.6615	16	44.8142	3.2	42.1836	44.011	53.04027	54.43409	16	42.79704
17	24.1768	3.4	24.4744	33.0698	17	44.8934	3.4	42.0322	44.2862	53.14856	53.77723	17	40.04744
18	25.6064	3.6	25.0354	32.5749	18	44.8015	3.6	42.2126	44.4634	53.44254	52.96865	18	38.90262
19	25.1287	3.8	25.6273	32.977	19	44.7982	3.8	42.8774	44.5452	57.05264	53.11857	19	30.56275
20	25.7518	4	30.6019	33.3856	20	44.9692	4	42.7516	44.4048	58.42633	56.64022	20	28.45307
21	25.8662	4.2	34.3403	32.9598	21	44.6899	4.2	42.3335	44.4855	58.68803	59.64714	21	1.28505
22	25.3985	4.4	33.3747	36.2625	22	43.8119	4.4	42.4523	44.4633	56.95335	59.05474	22	1.1324
23	25.9289	4.6	32.8291	39.0962	23	42.7569	4.6	42.843	44.6108	54.75494	59.19624	23	1.12452

24	25.9009	4.8	33.7816	41.1607	24	41.6287	4.8	42.8709	44.6004	55.13442	59.39034	24	1.03229
25	24.418	5	34.8671	41.1322	25	40.8956	5	43.2026	45.0925	56.28942	59.49353	25	0.96289
26	24.992	5.2	35.138	41.4302	26	40.5758	5.2	43.1418	45.3007	56.24382	59.43934	26	0.97472
27	24.2729	5.4	34.9441	40.9136	27	40.6736	5.4	42.8724	45.2638	55.79018	59.44536	27	0.99709
28	24.6784	5.6	34.7719	40.6531	28	41.0559	5.6	43.0787	45.1763	55.76189	59.54315	28	0.99831
29	25.8684	5.8	35.1446	41.0134	29	41.3024	5.8	44.7811	45.3304	56.56999	59.39722	29	0.90421
30	25.6669	6	35.3088	41.3347	30	41.7019	6	44.432	45.29	55.69881	59.47808	30	0.90456
31	25.9058	6.2	35.4879	41.4879	31	42.9928	6.2	44.0595	45.3125	55.57156	59.51965	31	0.90605
32	25.175	6.4	35.6821	41.7558	32	43.2912	6.4	44.7749	45.4586	58.14834	59.39769	32	0.91165
33	26.9644	6.6	35.8749	41.7704	33	43.3677	6.6	43.8814	45.4784	57.96232	59.48943	33	0.94382
34	27.3389	6.8	36.2081	41.8025	34	44.4522	6.8	44.4443	45.5659	55.78935	59.4964	34	0.89396
35	26.0557	7	36.2892	41.8231	35	44.3177	7	46.349	53.03346	55.38455	59.48958	35	0.90343
36	27.1967	7.2	36.4286	44.0017	36	44.6307	7.2	46.1856	53.6519	55.31103	59.69017	36	0.90585
37	27.7907	7.4	36.3986	43.6802	37	43.928	7.4	45.5044	54.01968	55.47404	59.54236	37	0.91817
38	27.0841	7.6	36.4245	43.6673	38	43.4587	7.6	45.1888	53.33596	55.45274	59.87957	38	0.90215
39	26.6729	7.8	36.6382	43.7571	39	42.9219	7.8	45.6539	53.84291	55.42818	59.73255	39	0.90011
40	26.5607	8	37.0332	43.618	40	42.8826	8	45.6476	53.45615	55.38225	59.86634	40	0.83811
41	26.3833	8.2	37.2617	43.8263	41	42.9037	8.2	45.7108	53.73792	55.16157	59.70789	41	0.89524
42	26.8169	8.4	37.3748	43.8574	42	42.6844	8.4	45.9002	53.6686	54.62283	59.84276	42	0.88166
43	27.4448	8.6	37.5231	43.9932	43	43.1758	8.6	46.1724	53.67741	54.68181	59.81994	43	0.77332
44	27.3466	8.8	37.6077	44.1339	44	43.2623	8.8	46.1655	53.63659	54.72368	59.81636	44	0.90031

45	27.6029	9	37.5742	44.0897	45	43.3872	9	46.1421	53.66012	54.69035	59.83174	45	0.81007
46	29.0046	9.2	37.5576	44.0883	46	43.4023	9.2	46.0496	53.68877	54.79361	59.85997	46	0.87361
47	29.8913	9.4	37.3539	44.173	47	42.934	9.4	45.9035	53.65423	55.12666	59.86185	47	0.83969
48	28.5513	9.6	37.4216	44.2973	48	42.4221	9.6	45.8462	53.63441	55.1167	59.85948	48	0.79145
49	28.4835	9.8	37.8179	44.3061	49	42.5458	9.8	45.7296	53.63471	55.24229	59.77672	49	0.8082
50	28.8008	10	37.8475	44.3017	50	42.5912	10	45.9137	53.79466	55.35324	59.81876	50	0.81077
51	29.3176	10.2	38.0394	44.1841	51	43.6369	10.2	45.8333	53.53741	55.78286	59.848	51	0.8884
52	28.595	10.4	38.068	44.1923	52	42.8733	10.4	45.9343	53.53912	55.77543	59.83161	52	0.80303
53	28.2126	10.6	38.023	44.259	53	42.286	10.6	45.9751	53.67428	55.68122	59.86493	53	0.83095
54	28.8104	10.8	37.7698	44.3685	54	42.8204	10.8	46.2242	53.72119	55.75439	59.83833	54	0.80373
55	28.3052	11	37.4433	44.4392	55	42.8196	11	46.2549	53.69849	55.78309	59.85157	55	0.82542
56	28.5617	11.2	37.6441	44.4226	56	42.3686	11.2	46.3067	53.6828	55.74263	59.79601	56	0.78673
57	28.4137	11.4	37.4924	44.3742	57	42.389	11.4	46.3097	53.77576	55.76151	59.88004	57	0.77945
58	27.6686	11.6	38.0065	44.3747	58	42.867	11.6	46.4841	53.67002	55.87516	59.86923	58	0.79858
59	29.3322	11.8	37.8561	44.2661	59	43.429	11.8	46.4725	53.75164	55.99668	59.81216	59	0.88657
60	27.9577	12	37.9394	44.1394	60	42.4777	12	46.7034	53.58052	55.90481	59.86869	60	0.76835
61	28.7965	12.2	37.995	44.0764	61	43.0899	12.2	46.7053	53.60048	55.79483	59.82367	61	0.83544
62	28.7864	12.4	38.0627	44.1276	62	42.852	12.4	46.5454	53.63585	55.89733	59.85912	62	0.8001
63	28.4836	12.6	38.0656	44.1848	63	43.3737	12.6	46.6794	53.71931	56.05659	59.8761	63	0.85074
64	28.1378	12.8	37.9834	44.1117	64	43.0339	12.8	46.8424	53.71647	56.7297	59.79703	64	0.88229
65	28.9963	13	37.843	44.1548	65	43.2315	13	46.9167	53.53223	56.27949	59.84416	65	0.83342

66	28.6475	13.2	37.6158	44.2402	66	43.4187	13.2	46.9068	53.58617	55.58158	59.85908	66	0.84484
67	29.8159	13.4	37.6873	44.2045	67	43.4315	13.4	46.8576	53.46785	55.94102	59.86727	67	0.80547
68	28.9611	13.6	37.4545	44.1789	68	43.1168	13.6	46.9145	53.55552	56.02027	59.87158	68	0.81802
69	29.7489	13.8	37.5966	44.2277	69	43.5453	13.8	47.0356	53.53805	56.00166	59.8296	69	0.81441
70	28.1798	14	37.7578	44.2595	70	43.1538	14	47.0687	53.56831	55.7881	59.87409	70	0.82307
71	27.9142	14.2	37.8866	44.2856	71	43.295	14.2	47.0128	53.5676	55.63527	59.87351	71	0.82294
72	27.1509	14.4	37.8436	44.4346	72	43.0578	14.4	47.0068	53.5826	55.76658	59.86435	72	0.79163
73	27.6151	14.6	38.0649	44.5054	73	42.7408	14.6	47.0717	53.59536	55.77847	59.8762	73	0.74582
74	28.3201	14.8	38.0392	44.4715	74	43.4785	14.8	47.1371	53.60223	55.59451	59.86075	74	0.74906
75	28.7909	15	38.0916	44.4501	75	43.1551	15	47.2817	53.60314	55.56547	59.87918	75	0.79816
76	27.9215	15.2	38.1198	44.399	76	43.2322	15.2	47.4256	53.60595	55.62289	59.87969	76	0.78194
77	28.753	15.4	38.417	44.4438	77	42.947	15.4	47.4789	53.60916	55.69211	59.84486	77	0.80101
78	28.5587	15.6	38.3805	44.4875	78	43.3126	15.6	47.5155	53.60296	55.67524	59.875	78	0.8518
79	29.857	15.8	38.2103	44.5665	79	42.9246	15.8	47.4768	53.55522	55.64121	59.86012	79	0.77391
80	32.5976	16	38.2958	44.556	80	43.0138	16	47.3602	53.57834	55.62501	59.8712	80	0.78522
81	30.1834	16.2	38.2794	44.4853	81	42.7151	16.2	47.3475	53.5968	55.687	59.87836	81	0.77006
82	28.7461	16.4	38.5234	44.4048	82	42.7877	16.4	47.3887	53.5957	55.83621	59.85555	82	0.80015
83	30.1987	16.6	38.7829	44.3916	83	42.5693	16.6	47.3868	53.5737	55.84331	59.88087	83	0.8137
84	31.5269	16.8	38.9175	44.3817	84	42.495	16.8	47.3866	53.56453	55.81282	59.87037	84	0.82077
85	31.9191	17	38.6929	44.4086	85	43.2422	17	47.3618	53.58126	55.83755	59.87869	85	0.77724
86	30.5847	17.2	38.6789	44.4297	86	43.096	17.2	47.31	53.55396	55.82978	59.88107	86	0.82602

87	31.6998	17.4	38.6088	44.3748	87	42.72	17.4	47.2208	53.55781	55.81805	59.87636	87	0.80557
88	31.7916	17.6	38.6742	44.4141	88	43.8231	17.6	47.182	53.63078	55.82287	59.8809	88	0.81504
89	32.0565	17.8	38.8995	44.3638	89	43.4723	17.8	47.2378	53.56526	55.80547	59.85516	89	0.84588
90	30.8039	18	38.933	44.295	90	43.6605	18	47.1972	53.62151	55.85188	59.87445	90	0.78485
91	32.866	18.2	39.181	44.2746	91	43.372	18.2	47.1594	53.56779	55.77165	59.88129	91	0.83736
92	33.0544	18.4	39.1978	44.2316	92	43.3113	18.4	47.0402	53.62488	55.7357	59.87704	92	0.76774
93	29.2831	18.6	39.0255	44.2375	93	42.9412	18.6	47.0853	53.55521	55.77153	59.88052	93	0.76865
94	33.1824	18.8	38.8277	44.3278	94	44.2156	18.8	47.1023	53.57765	55.83497	59.86716	94	0.81512
95	32.4821	19	39.0469	44.342	95	42.9593	19	47.2193	53.62362	55.79436	59.8752	95	0.81792
96	31.1053	19.2	39.0505	44.31	96	43.055	19.2	47.2313	53.62247	55.76023	59.87308	96	0.77809
97	32.5368	19.4	39.0968	44.2949	97	43.3271	19.4	47.1326	53.65288	55.84118	59.88027	97	0.76944
98	32.7775	19.6	38.9365	44.2727	98	42.7926	19.6	46.9712	53.6801	55.85004	59.87527	98	0.78817
99	31.7	19.8	38.8966	44.2959	99	43.3863	19.8	47.0658	53.63362	55.6964	59.88153	99	0.79066
100	29.9487	20	38.9716	44.3113	100	42.6573	20	47.2019	53.64269	55.63927	59.88153	100	0.82314
